# Computational analysis of arrhythmogenesis in *KCNH2* T618I mutation-associated short QT syndrome and the pharmacological effects of quinidine and sotalol

**DOI:** 10.1038/s41540-022-00254-5

**Published:** 2022-11-04

**Authors:** Shugang Zhang, Weigang Lu, Fei Yang, Zhen Li, Shuang Wang, Mingjian Jiang, Xiaofeng Wang, Zhiqiang Wei

**Affiliations:** 1 College of Computer Science and Technology, Ocean University of China, Qingdao, 266100 China; 2 Department of Educational Technology, Ocean University of China, Qingdao, 266100 China; 3 Biological Physics Group, School of Physics and Astronomy, The University of Manchester, Manchester, M13 9PL UK; 4 School of Mechanical, Electrical, and Information Engineering, Shandong University, Weihai, 264200 China; 5 College of Computer Science and Technology, Qingdao University, Qingdao, 266071 China; 6 College of Computer Science and Technology, China University of Petroleum (East China), Qingdao, 266580 China; 7 School of Information and Control Engineering, Qingdao University of Technology, Qingdao, 266033 China; 8 MindRank AI ltd., Hangzhou, Zhejiang, 311113 China

**Keywords:** Computational biology and bioinformatics, Cardiology

## Abstract

Short QT syndrome (SQTS) is a rare but dangerous genetic disease. In this research, we conducted a comprehensive in silico investigation into the arrhythmogenesis in *KCNH2* T618I-associated SQTS using a multi-scale human ventricle model. A Markov chain model of *I*_Kr_ was developed firstly to reproduce the experimental observations. It was then incorporated into cell, tissue, and organ models to explore how the mutation provided substrates for ventricular arrhythmias. Using this T618I Markov model, we explicitly revealed the subcellular level functional alterations by T618I mutation, particularly the changes of ion channel states that are difficult to demonstrate in wet experiments. The following tissue and organ models also successfully reproduced the changed dynamics of reentrant spiral waves and impaired rate adaptions in hearts of T618I mutation. In terms of pharmacotherapy, we replicated the different effects of a drug under various conditions using identical mathematical descriptions for drugs. This study not only simulated the actions of an effective drug (quinidine) at various physiological levels, but also elucidated why the *I*_Kr_ inhibitor sotalol failed in SQT1 patients through profoundly analyzing its mutation-dependent actions.

## Introduction

Short QT syndrome (SQTS) is an inherited and life-threatening cardiac channelopathy characterized by an abnormally short QT interval and increased risks for cardiac arrhythmias and sudden cardiac deaths (SCD). Depending on the mutated genes, the SQTS variants are named SQT1-8 and correlated with various ion channels, including potassium channels (SQT1-3)^1–5^, calcium channels (SQT4-6)^6,7^, sodium channels (SQT7)^8^ and anion exchangers (SQT8)^9^. Among these variants, SQT1 is the one that presents in the majority of patients, being responsible for up to 15% of all cases^10^. The SQT1 arises from gain-of-function mutations in the *KCNH2* gene, and was first identified in 2004 by Brugada et al. with a substitution mutation N588K^1^. In 2010, another mutation in the *KCNH2* gene was reported in a Chinese family that the threonine at carbon 618 was replaced by an isoleucine (i.e., T618I)^11^. The ECG screening showed a strong family history of SCD, and characteristics of SQTS including abbreviated QT intervals and peaked T-waves were also observed on the ECG. Additional electrophysiological tests revealed that the *I*_Kr_ was markedly increased in the T618I mutant channel, which was mainly caused by the significantly altered inactivation curve that shifted almost 50 mV towards the depolarized direction^11^. Despite the above clinical and genetic findings, mechanisms regarding how these molecular changes finally lead to a disastrous malignant arrhythmia remain to be elucidated.

To explore the proarrhythmic effects of the *KCNH2* T618I mutation, an in silico investigation was conducted in this study based on a multi-scale virtual heart. First, a biophysically-accurate and validated Markov chain model for *I*_Kr_ was developed to recapitulate the experimental observations at the subcellular level. The developed *I*_Kr_ model was then incorporated into a well-established human ventricle model to determine the functional consequences of the T618I mutation on the action potential (AP) and the transmural heterogeneity of repolarization. Next, the arrhythmogenic substrate in the T618I mutation condition was investigated using tissue models. The sustainability of arrhythmias was also evaluated using realistic 2D ventricular slice and organ models. Finally, in terms of pharmacotherapy, we investigated the potential therapeutic effects of quinidine, a clinically available drug, by incorporating its known interactions with multiple ion channels. We also simulated the actions of *I*_Kr_ inhibitor sotalol and analyzed why it failed in SQT1 patients.

## Results

### Simulation results of the remodeling effects of *KCNH2* T618I mutation

#### Simulation of *I*_Kr_ in wild-type and *KCNH2* T618I mutation conditions

**Fig. 1:**
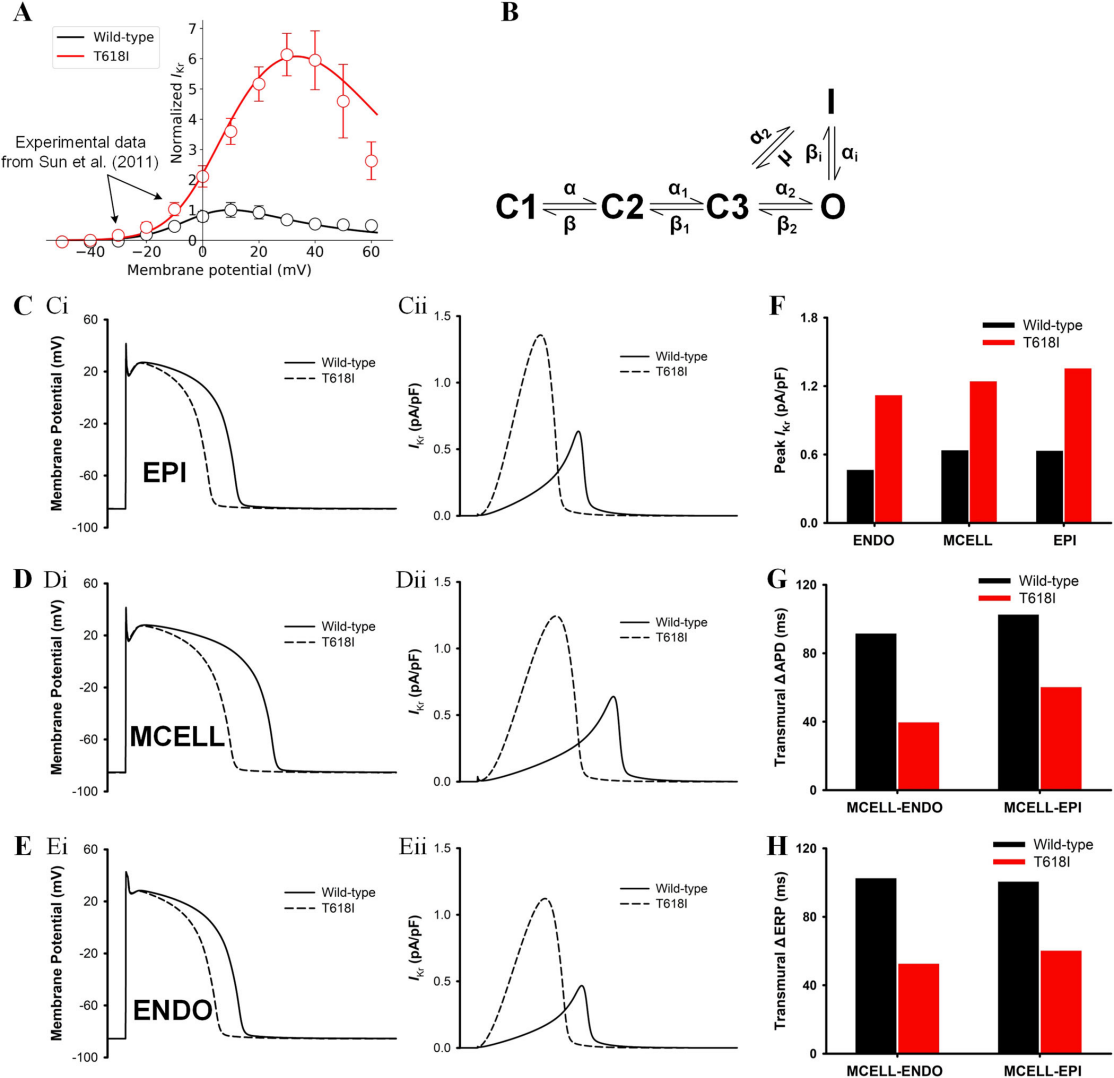
Simulation results at ion channel and cellular levels. **A** The fitted current–voltage (I–V) curves in wild-type (black) and T618I (red) conditions using *I*_Kr_ Markov chain models. Experimental data from Sun et al.^11^ (their Fig. 4B). Error bars represent the standard error of measurement (SEM). **B** The *I*_Kr_ Markov chain model. **C** Steady-state (1.25 Hz) action potentials (Ci) and the corresponding *I*_Kr_ (Cii) for EPI cells. Solid and dashed lines represent wild-type and T618I conditions, respectively. **D** Steady-state action potentials and the corresponding *I*_Kr_ for MIDDLE cells. **E** Steady-state action potentials and the corresponding *I*_Kr_ for ENDO cells. **F** The comparison of the peak *I*_Kr_ in wild-type and T618I conditions. **G** The comparison of transmural APD differences. **H** The comparison of transmural ERP differences.

The developed *I*_Kr_ Markov chain model was evaluated by comparing the generated I–V curve with the experimental data from Sun et al.^11^ (their Fig. 4B). Both the experimental data and the simulated I–V curve were normalized to their corresponding maximum currents (around 10 mV) under the wild-type condition, as shown in Fig. 1A. It can be observed that the model-generated I–V curve fitted well with the experimental data in AP-relevant membrane potential range, and the two main phenotypes of the mutant *I*_Kr_ were successfully recapitulated by the model, namely the right-shifted I–V relationship and the greatly enhanced *I*_Kr_ amplitude. These results suggested that the developed model was able to simulate the effects of the T618I mutation at the ion channel level.

#### Effects of the *KCNH2* T618I mutation on action potentials

The developed *I*_Kr_ Markov chain model was then incorporated into the Tusscher-Noble-Noble-Panfilov (TNNP06) model to explore the effects of the *KCNH2* T618I mutation at the cellular level. Steady-state APs of different cell types in wild-type and T618I conditions, as well as their corresponding *I*_Kr_, are presented in Fig. 1. Simulation results suggested that the T618I mutation accelerated the repolarization phase of action potentials through enhancing *I*_Kr_, which led to abbreviated ventricular APD and ERP.

Though ERP was shortened in all cell types, this effect was not the same in different cell types, with MCELL being the most affected one among three cell types. The differently reduced APD also led to the remodeling of transmural dispersion of repolarization. The membrane potential difference of APD (ΔAPD) between ENDO and MCELL was decreased by almost 60% from 91.7 to 39.7 ms, and the ΔAPD between EPI and MCELL was also reduced from 102.7 to 60.3 ms. Likewise, transmural differences of ERP (ΔERP) were decreased accordingly (Fig. 1G, H).

#### Influences of the *KCNH2* T618I mutation on APD and CV restitution properties and the rate adaption of QT interval

**Fig. 2:**
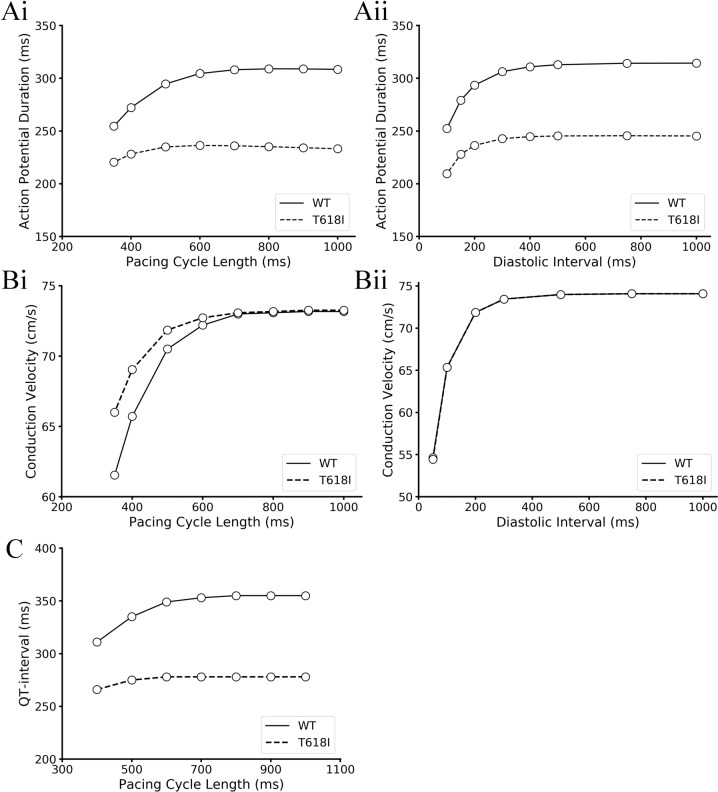
Simulation results of APD and CV restitution curves and rate adaption curve of QT interval. **A** APD restitution curves obtained from the S1–S2 protocol (**Ai**) and the dynamic protocol (**Aii**), where APDs were plotted against PCL and DI, respectively. **B** CV restitution curves obtained from the S1–S2 protocol (**Bi**) and the dynamic protocol (**Bii**). **C** Rate adaption curve of QT interval.

Clinical reports have demonstrated a reduced rate adaption of QT in SQTS patients during exercise tests^12^. To investigate the mechanism underlying the reduced rate adaption of QT interval, we conducted a series of simulations regarding the restitution properties using standard S1–S2 and dynamic protocols. Depending on the level at which the experiments were performed, these experiments can be organized as APD restitution curve (cellular level), conduction velocity restitution curve (tissue level), and rate adaption curve of QT interval (organ level). Simulation results are illustrated in Fig. 2.

First, for the cellular level APD restitution properties, it can be observed in Fig. 2Ai, Aii that the APD restitution curves under mutation conditions obtained from either S1–S2 protocol or dynamic protocol showed shallower slopes compared with wild-type conditions, indicating a reduced rate adaption of APD at higher heart rates. Next, for the CV restitution curve at the tissue level, the two protocols showed diverse results. Specifically, under the setting of dynamic protocol, restitution curves in the mutation and wild-type conditions diverged when the PCL was below 700 ms. The conduction velocity of excitation waves in the mutant tissue strand was larger than that in the wild-type tissue at smaller PCLs. In contrast, the S1–S2 protocol generated identical curves (Fig. 2Bii) for these two conditions. The two different measuring results suggested that the change of CV depended on the diastolic interval, and the mutation increased the CV indirectly via the abbreviation of APD. Finally, at the organ level, simulation results demonstrated a shortened QT interval irrespective of heart rate (Fig. 2C). However, the QT discrepancy between the two conditions became smaller at faster heart rates (i.e., shorter PCLs), which was caused by the impaired rate adaption of QT interval in the mutant tissue. In particular, the mutant tissue demonstrated a shallower slope compared with WT, and this phenomenon was in line with the cellular observation that the APD of mutant myocyte also showed a reduced rate adaption.

#### Effects of the *KCNH2* T618I mutation on the temporal vulnerability to the unidirectional conduction block

**Fig. 3:**
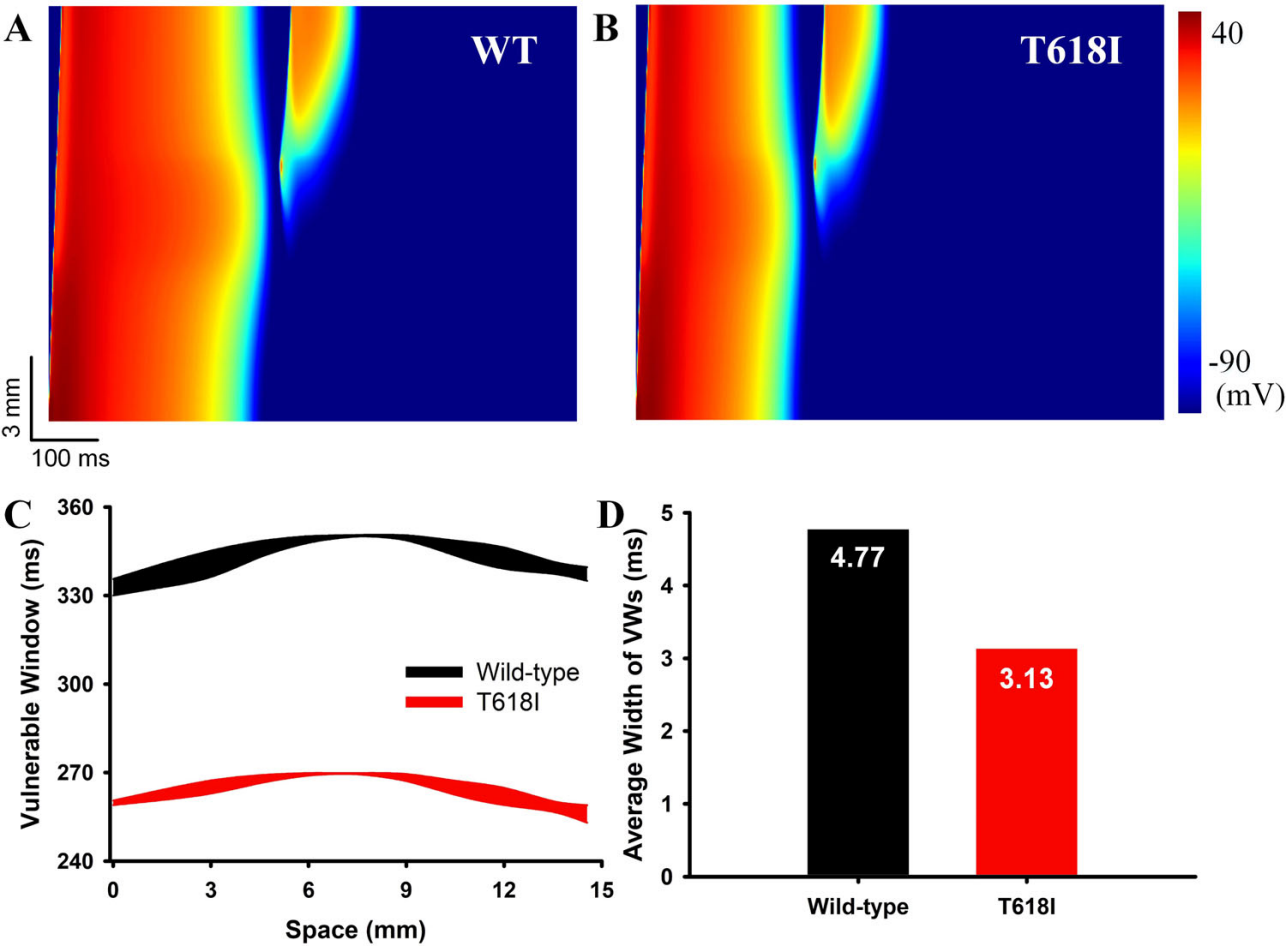
Simulation results of the unidirectional conduction block using the transmural 1D strand. **A** The evoked unidirectional conduction block in the wild-type condition. **B** The unidirectional conduction block in the T618I mutation condition. **C** Distributions of VWs in the two conditions. **D** The comparison of the average VW width in the two conditions.

To investigate the influences of the T618I mutation on tissue’s susceptibility to the unidirectional conduction block, the vulnerable window (VW) was measured separately in wild-type and mutant transmural strands. Figure 3 presents the simulated unidirectional conduction block phenomenon in wild-type and mutation conditions at a position of 7.5 mm away from the endocardial end. It can be observed that the evoked excitation wave propagated unidirectionally towards the epicardial end due to a shorter refractory period of the epicardium. The measured VW width at the specific position was 1.7 ms (from 348.7 to 350.4 ms) in wild-type, and it was enlarged to 2.4 ms (from 267.1 to 269.5 ms) in the T618I condition, indicating a higher temporal vulnerability in the mutant tissue at this position. However, further measurements of other locations on the strand suggested that the overall temporal vulnerability was actually decreased in the mutation condition, and the increased VW was only observed in locations within a local 2-mm segment (approximately from 7.5 to 9.5 mm). The overall distribution of VWs on the strand, and the average VW width in wild-type and T618I conditions, are plotted in Fig. 3C, D.

#### Effects of the *KCNH2* T618I mutation on the spatial vulnerability to reentry arrhythmias

**Fig. 4:**
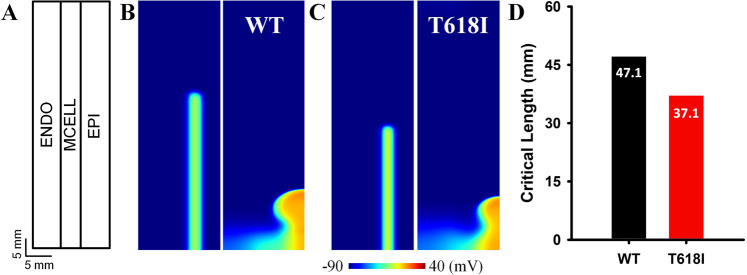
Simulation results of the critical length in the idealized 2D sheet. **A** The schematic diagram of the idealized 2D sheet. **B** The critical length of S2 for initiating spiral waves in the wild-type tissue. **C** The critical length in the T618I mutant tissue. **D** The comparison of critical length in the two situations.

The influences of the *KCNH2* T618I mutation on the spatial vulnerability to reentry arrhythmias were evaluated by measuring the critical length (CL) in an idealized 2D sheet. The critical length provided a quantitative index for the inducibility of reentry arrhythmia in terms of spatial perspective, and a shorter CL indicated that the tissue was more prone to reentry arrhythmias. The CL was related to the wavelength (the product of ERP and CV) of excitation. In the simulation, the measured CL was up to 47.1 mm in the wild-type condition (Fig. 4B) due to a comparatively long ERP of the wild-type myocytes. In contrast, the CL was 37.1 mm in the mutation condition (Fig. 4C), decreased by almost a quarter comparing to the wild-type tissue. This was due to the accelerated repolarization and abbreviated ERP resulted from the enhanced *I*_Kr_ in the T618I condition.

#### Effects of the *KCNH2* T618I mutation on the dynamic behavior of reentrant spiral waves

To investigate the influences of the T618I mutation on the dynamic behavior of reentrant spiral waves such as the rotation frequency and sustainability, simulation experiments were conducted using 2D and 3D models. First, we initiated spiral waves using S1–S2 protocols in an idealized tissue sheet (75 × 75 mm^2^) to investigate the influences of the mutation on rotation behavior of spiral waves. Simulation results demonstrated that both conditions produced stable spiral waves and tip trajectories (see Supplementary Fig. 4). However, a significant decrease in wavelength was observed under the mutation condition, and the rotation frequency was increased from 4.08 to 4.55 Hz.

**Fig. 5:**
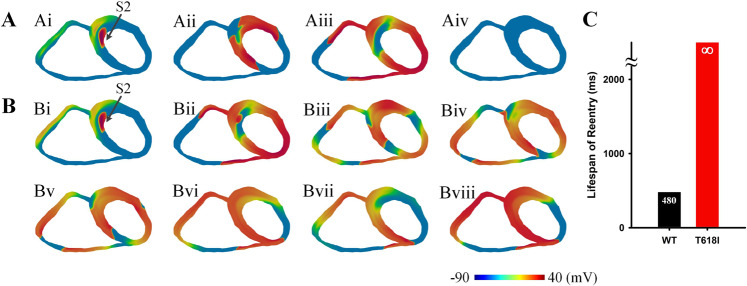
Simulation results of reentry arrhythmias in the realistic 2D ventricular slice. Spiral waves were evoked by the S1–S2 protocol. The stimulating locations of S2 were indicated by the black arrows. **A** Spiral waves in the wild-type condition with a lifespan of only 480 ms. Snapshots were recorded at 320, 390, 490, 800 ms. **B** The spiral wave in the T618I mutation condition. Snapshots were recorded at 260, 410, 1090, 1760, 1940, 2330, 2630, 2930 ms. Spiral waves took the form of functional reentry initially before it transformed into anatomical reentry arrhythmia at about 2000 ms and persisted since then. **C** The comparison of lifespan of spiral waves in two situations.

Next, we investigated the influences of T618I mutation on the sustainability of reentry arrhythmias. Simulation experiments were performed on realistic models, i.e., the 2D ventricular slice and 3D bi-ventricle. The S1–S2 protocol was used to initiate the reentrant excitation waves. The simulated reentrant spiral waves on 2D tissue slices for T618I and wild-type conditions were separately demonstrated in Fig. 5A, B. In the mutation condition, the evoked spiral wave circulated around a functionally determined core and lasted 1740 ms with more than 8 cycles. The functional reentry then transformed into anatomical reentry arrhythmia and persisted since then. In contrast, the spiral wave evoked in the wild-type slice was unsustainable, and it self-terminated with a brief lifespan. This was due to the relatively long wavelength of the wild-type spiral wave that could hardly be accommodated within a limited tissue size.

**Fig. 6:**
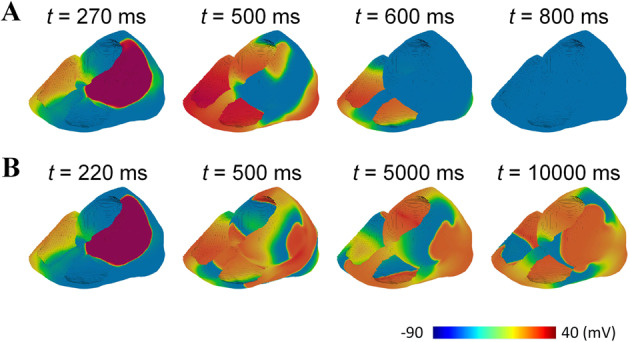
Simulation results of reentry arrhythmias in the realistic 3D bi-ventricle model. The S2 stimulus was applied to the upper part of the LV epicardium to initiate scroll waves. **A** The scroll wave in the wild-type condition. The lifetime was only 410 ms, and the recorded onset and disappearance time of the scroll wave were separately 270 and 680 ms. **B** The scroll wave in the T618I mutation condition, which persisted throughout the whole simulation period of 10,000 ms.

The above observations in 2D slices were further verified on the realistic 3D bi-ventricle model. The simulated scroll waves in the 3D bi-ventricle models under wild-type and mutation conditions were illustrated in Fig. 6. It can be observed that, in both wild-type and mutation conditions, a premature stimulus applied on the upper part of the left ventricle during the vulnerable window could successfully evoke a unidirectional conduction block, which then transformed into scroll waves in the 3D ventricle. However, the evoked scroll wave in the wild-type ventricle was not sustainable and disappeared after 800 ms (see Fig. 6B). In contrast, the evoked scroll wave in the T618I mutant ventricle persisted throughout the whole simulation period of 10,000 ms, with its functional core anchoring the side wall of the left ventricle.

### Simulation results of the actions of quinidine

#### Actions of quinidine on action potentials of *KCNH2* T618I mutation

**Fig. 7:**
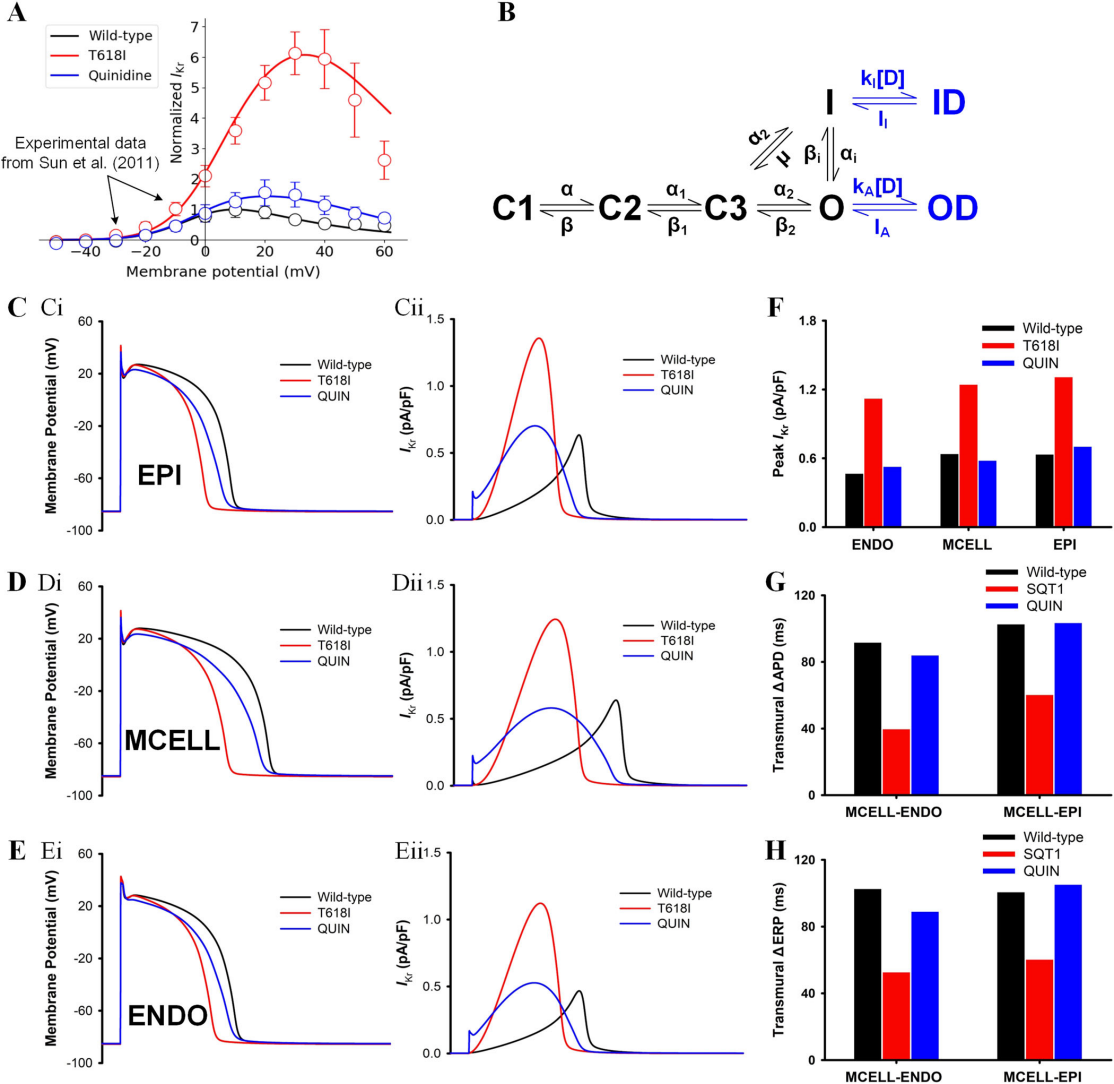
Actions of quinidine at ion channel and cellular levels. **A** The fitted current–voltage (I–V) curves in wild-type (black), T618I (red), and quinidine (blue) conditions using *I*_Kr_ Markov chain models. Experimental data from Sun et al.^11^ (their Fig. 7B). Error bars represent the standard error of measurement (SEM). **B** The *I*_Kr_ Markov chain model with two additional drug-related states. **C**–**E** Steady-state (1.25 Hz) action potentials and the corresponding *I*_Kr_ for EPI, MID, and ENDO cells. **F** The comparison of the peak *I*_Kr_. **G** The comparison of transmural APD differences. **H** The comparison of transmural ERP differences.

To investigate the pharmacological mechanisms of quinidine (i.e., a common antiarrhythmic drug for treating SQTS) on the *KCNH2* T618I-associated short QT syndrome, we modeled the drug actions by adding two additional states to the Markov chain states (Fig. 7B) as in Whittaker et al.’s study^13^. Simulation results at ion channel and cellular levels after the addition of 5 μmol quinidine, along with that under control and mutation conditions, are gathered in Fig. 7.

First, it can be observed in Fig. 7A that the I–V curves generated by the Markov model fitted well with the experimental data from Sun et al.^11^ (their Fig. 7B). Both the experimental data and the simulated I–V curve were normalized to their corresponding maximum currents under the WT condition. The well-fitted I–V curves under various conditions illustrated the rationality of the developed Markov model. In terms of APD, quinidine reversed the abbreviated APD in all cell types (Fig. 7Ci, Di, Ei), and such effect was attributed to the suppressed *I*_Kr_. As plotted in Fig. 7Cii, Dii, Eii, F, quinidine reduced the *I*_Kr_ almost to its control level. Therefore, the increased repolarizing forces in mutation conditions were attenuated and thereby the APD was restored.

In line with APD, the ERP was also restored after the application of quinidine. In detail, the ERP of EPI and ENDO cells increased by 38.6 and 47.5 ms, respectively, while for MID cells the ERP increased significantly by 83.76 ms. The reversed refractory periods of three cell types in the drug group were comparable to their control levels. In terms of the transmural dispersion of repolarization, ΔAPD and ΔERP were also restored to their control levels (Fig. 7G, H).

**Fig. 8:**
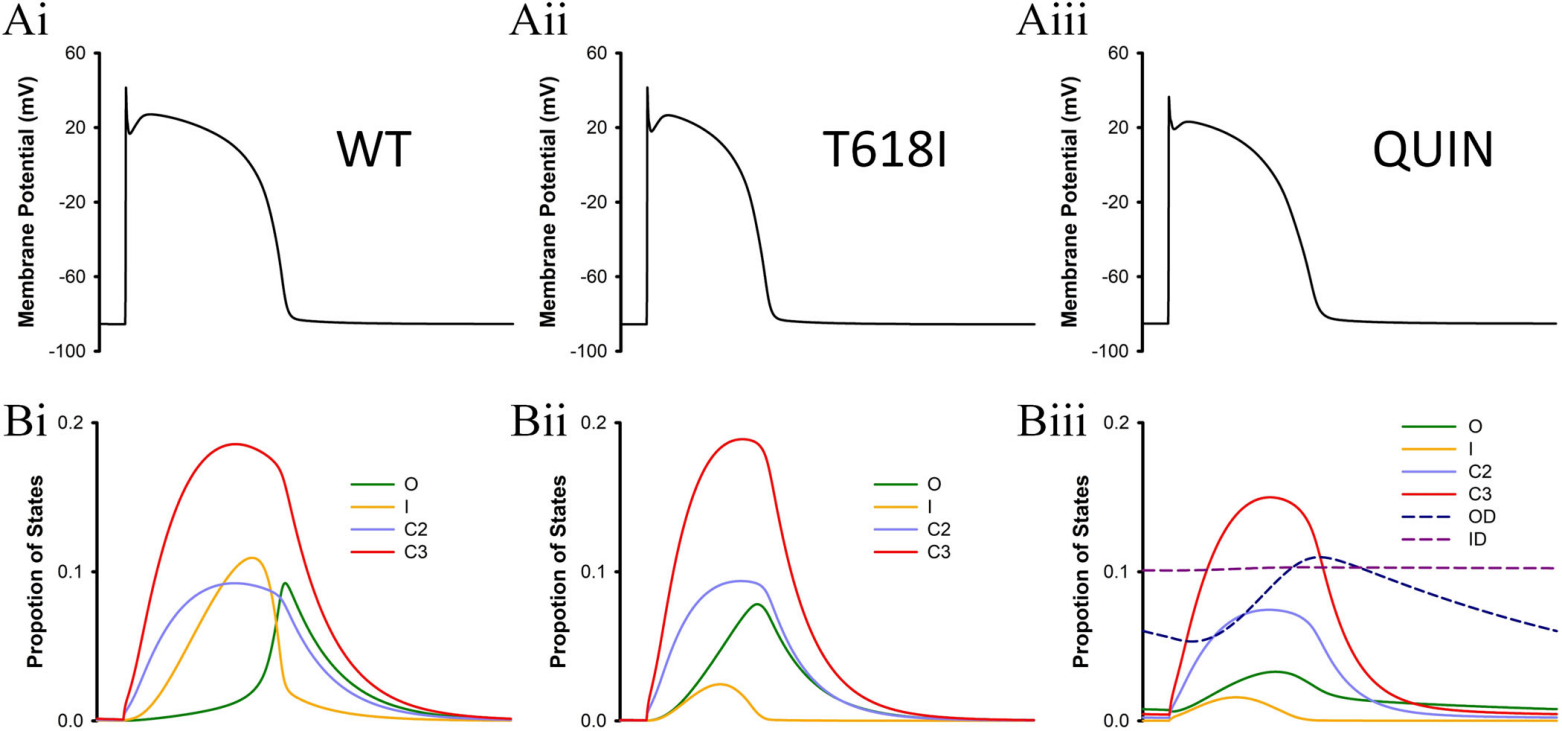
Proportion of states under WT, T618I, and QUIN conditions. **A** Action potential traces. **B** The corresponding state proportions under different conditions. The Markov model demonstrated that the enhanced *I*_Kr_ in T618I was due to the loss of inactivation, as evidenced by the significantly dropped proportion of the Inactivation (“I”) state in panels **Bi** and **Bii**. Simulation also showed that quinidine exerted its effects by directly deprecating the Open (“O”) state rather than restoring the “I” state (see panels **Bii** and **Biii**).

To give further insights into the pharmacological actions of quinidine at the subcellular level, we plot the proportion of states under different conditions, as shown in Fig. 8.

It can be observed in Fig. 8Bi, Bii that there was not much difference in the peak proportion of the Open state between WT and T618I conditions. However, the Open state started earlier in T618I and therefore corresponded to an overall larger current comparing to the WT condition. By further comparing the inactivation state of the two conditions, it can be found that the Inactivation state in the mutation condition was significantly lower than that in WT. Therefore, the enhanced *I*_Kr_ was due to the early opening of the *I*_Kr_ channel, which in turn arose from the loss of inactivation by the T618I mutation. In addition, the simulation results of quinidine (Fig. 8Biii) suggest that the drug did not exert its effects by restoring the lost inactivation in T618I cells, as evidenced by the low level of the Inactivation state. Instead, quinidine suppressed the Open state directly by transferring it into drug-bound states (i.e., *OD* and *ID*).

#### Actions of quinidine on restitution properties and the rate adaption of QT interval

**Fig. 9:**
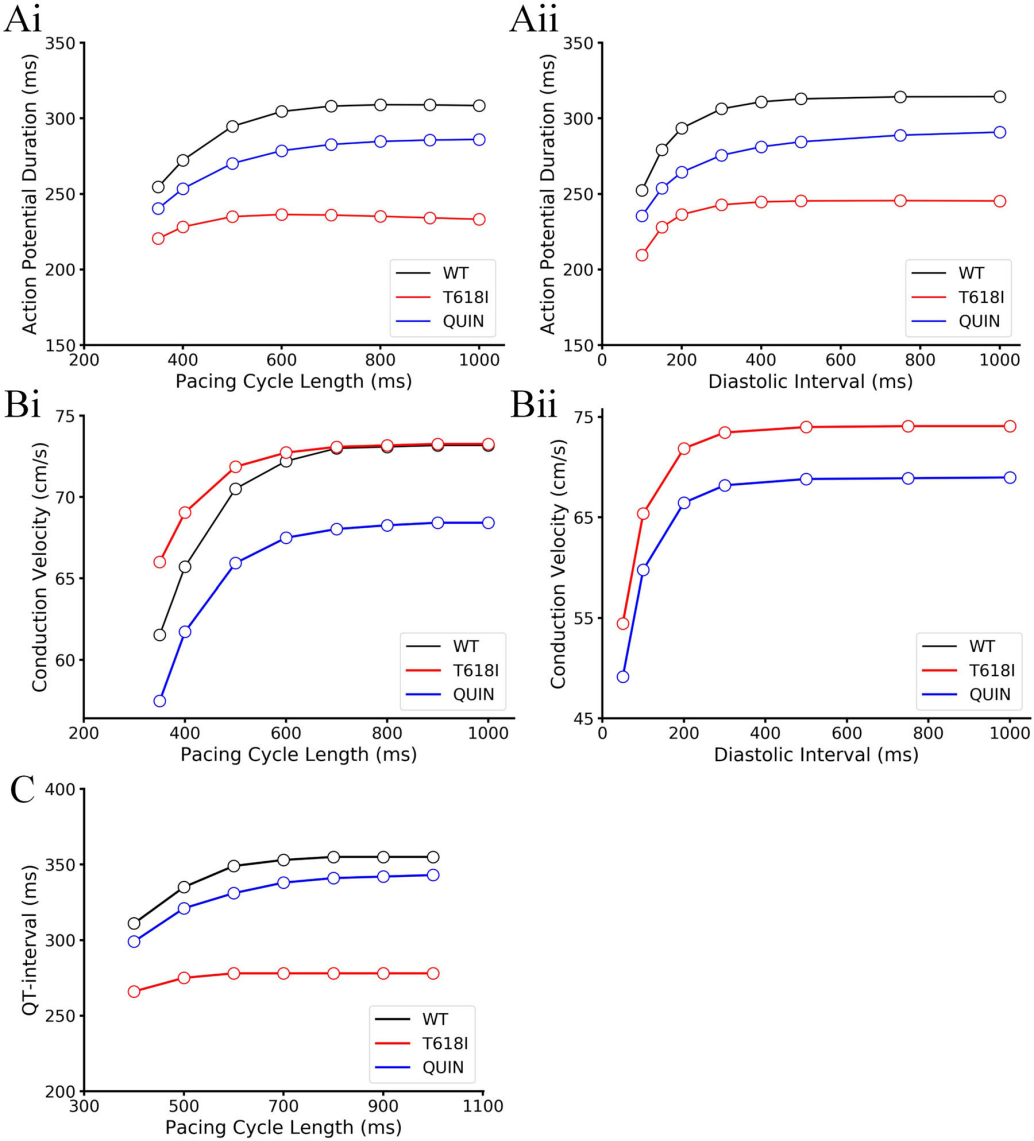
Simulation results of APD and CV restitution curves and the rate adaption curves of QT interval under wild-type, T618I mutation, and quinidine conditions. **A** APD restitution curves obtained from the S1–S2 protocol (**Ai**) and the dynamic protocol (**Aii**), where APDs were plotted against PCL and DI, respectively. **B** CV restitution curves obtained from the S1–S2 protocol (**Bi**) and the dynamic protocol (**Bii**). **C** The rate adaption curves of QT interval.

We next tested the drug efficacy in reversing the restitution properties and the rate adaption of QT interval that altered by the T618I mutation. In line with the wild-type and the mutation groups, both standard S1–S2 and dynamic protocols were adopted to generate restitution curves of APD, CV, and QT. Simulation results are illustrated in Fig. 9. Restitution curves under wild-type and mutation conditions were also plotted for comparison purposes.

Simulations demonstrated that quinidine partially restored the AP adaption at higher pacing rates under mutation conditions, producing a re-steepened restitution curve (Fig. 9Ai, Aii). For the CV restitution curve, quinidine led to a significant decrement of CV, and this influence was observed in both CV-PCL and CV-DI curves. The CV reduction effect of quinidine arose from its inhibitory action on sodium channels. More concretely, quinidine decreased *I*_Na_ by 21.3% at a dose of 5 μmol in our model. Finally, as shown in Fig. 9C, quinidine rescued the rate adaption of QT interval impaired by the T618I mutation. It also prolonged the QT interval in a significant manner regardless of the pacing rate.

#### Actions of quinidine on the spatial vulnerability to reentry arrhythmias

As suggested in previous sections, the spatial vulnerability was increased in the T618I mutation condition because of the shortened critical length for initiating reentrant excitation waves. In this regard, we investigated the effects of quinidine to explore whether it would reverse the increased spatial vulnerability. Simulation results were plotted in Supplementary Fig. 16. It can be observed that, quinidine greatly extended the CL from 37.1 to 56.6 mm and even exceeded that in the wild-type tissue (47.1 mm). The extended CL indicated the role of quinidine in decreasing spatial vulnerability, and this effect was mainly attributed to the ERP prolonging effects of quinidine through the inhibition of repolarization currents (*I*_Kr_, *I*_Ks_, *I*_K1_) and *I*_Na_.

#### Antiarrhythmic actions of quinidine in terms of the dynamic behavior of reentrant spiral waves

The antiarrhythmic effects of quinidine were also investigated by simulating its actions on the dynamic behavior of reentrant spiral waves. The aforementioned simulation results have demonstrated that the T618I mutation could lead to an increase in the rotation frequency (from 4.08 to 4.55 Hz) of induced spiral waves but without obvious change in the meandering size of the spiral wave tip. After the administration of quinidine (see Supplementary Fig. 5), the tip trajectory still did not show any significant change, but the shortened wavelength caused by the mutation was partially restored. Regarding the rotation frequency, quinidine also exerted antiarrhythmic effects by decreasing the frequency to 3.85 Hz.

**Fig. 10:**
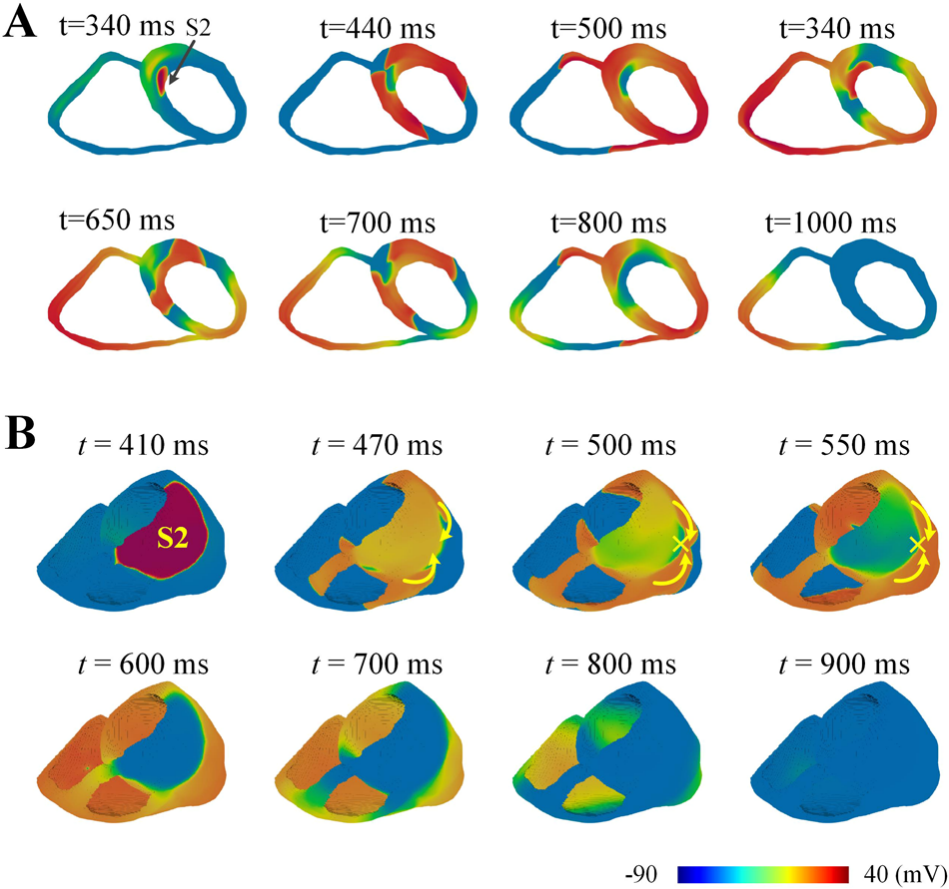
Simulation results of the reentry arrhythmias in the condition of quinidine. **A** Spiral waves on the 2D ventricular slice. Spiral waves were evoked by the S1–S2 protocol, and the black arrow indicates the stimulating location of S2. Quinidine prevented the persistent arrhythmias, which was evidenced by the disappearance of spiral waves at about 1000 ms. **B** Scroll waves in the 3D ventricle. In line with the 2D simulation results, the evoked scroll waves were not sustainable and disappeared at about 900 ms.

In addition to the rotation behavior of spiral waves, the antiarrhythmic effects of quinidine were also evaluated in terms of the sustainability of reentrant arrhythmias. Figure 10A presents the simulated reentrant spiral waves on the 2D tissue slice after administration of quinidine. Experiments showed that the lifespan of the induced spiral waves was only 790 ms, which indicated that quinidine could successfully prevent the sustained reentrant arrhythmias in the T618I mutation condition. In line with the 2D simulation results, the evoked scroll waves (Fig. 10B) were not sustainable in the drug group due to the prolonged refractory distance by quinidine. The scroll waves disappeared at around 900 ms.

#### Parallel simulations using two other models

To prove the robustness of the simulation observations reported in this paper and avoid potential model-dependent results, we conducted additional parallel simulations (i.e., identical experiments but with different cell models) using two other models, namely the O’hara-Rudy dynamic model (the ORd model^14^) and a contemporary one named ToR-ORd^15^. We followed a strict experimental procedure to ensure that all the parallel experiments based on ORd and ToR-ORd models were identical to that based on the TNNP06 model. Detailed simulation results using these two cell models were summarized in the Supplementary material. In general, the three models produced highly consistent results under both pathological and pharmacological conditions.

## Computational analysis of the poor pharmacological effects of sotalol in SQT1

Previous simulation studies have reported several drugs that may act therapeutic effects^13,16^. However, few literatures focused on those “failed” drugs, which were supposed to but actually failed to act therapeutic effects. For example, sotalol, a class III antiarrhythmic drug, was known to block *I*_Kr_ under normal conditions, but clinical reports have substantially vetoed its therapeutic effects in both T618I and N588K SQT1 patients^17,18^. To give a comprehensive analysis of the failure reasons of sotalol in different SQT1 variants, we constructed another Markov model that specifically for N588K, and then developed the drug-bound *I*_Kr_ model for sotalol and compared its actions under WT, T618I, and N588K conditions.

### Simulation of *I*_Kr_ in the *KCNH2* N588K mutation condition

Simulated I–V curves of the N588K, along with the experimental data from^1^, are illustrated in Supplementary Fig. 17. It can be observed that, compared with the T618I mutation, the N588K mutation did not exhibit significant inactivation over the physiological voltage range (−80~20 mV). Actually, simulations showed that the inactivation did not develop until the voltage reached approximately 55 mV. In contrast, while the voltage range over which inactivation of the T618I *I*_Kr_ occurred was also above the physiological range, it was shifted to a smaller extent than the N588K mutation^11^.

### Actions of sotalol on *I*_Kr_ under WT, T618I, and N588K conditions

We then developed the model of sotalol and compared its actions under WT, T618I, and N588K conditions. The simulated I–V curves under multiple doses of sotalol are illustrated in Supplementary Fig. 18.

It can be observed from Supplementary Fig. 18B, C that, at a clamped voltage of +20 mV, 100 μmol sotalol depressed WT *I*_Kr_ extensively by 49.4%, but only by 7.5% under the N588K condition. Even 500 μmol sotalol suppressed N588K *I*_Kr_ only by 29.7%. These simulation results match well with experimental observations (decreased by 48% for WT, 9.0% for N588K+ 100 μmol sotalol, and 27.0% for N588K+ 500 μmol sotalol^1^). Another phenomenon is that the inhibition ability of sotalol was significantly restored at more positive voltages, and the gap of drug efficacy between N588K and WT was reduced.

Due to the smaller shift of inactivation in T618I than N588K, the effect of sotalol is also different from that in N588K. Specifically, compared with the decrement of only 29.7% in N588K, 500 μmol sotalol could decrease the mutant *I*_Kr_ by 63.8% in the T618I condition, suggesting that the drug efficacy was less diminished in the latter case. This is in accordance with Sun et al.’s reports that 500 μmol sotalol was able to decrease T618I *I*_Kr_ substantially and such inhibitory effect was comparable to that achieved by 5 μmol quinidine.

We did not conduct more simulations in high-dimensional models since the cell model is enough to demonstrate the failure reasons of sotalol. On the other hand, most high-dimensional simulation results of sotalol can be expected. For example, the critical length would barely change even under 100 μmol sotalol.

## Discussion

### Main findings

Short QT syndrome is associated with high risks for life-threatening events. In this study, we investigated the proarrhythmic effects of the *KCNH2* T618I mutation using a multi-scale human virtual heart. The major findings of this study are as follows: (i) The T618I mutation unevenly shortened the ventricular APD and ER and decreased the transmural dispersion of repolarization, leading to a decreased susceptibility to arrhythmias in terms of temporal domain. (ii) The T618I mutation shortened the tissue excitation wavelength through a reduction of the ERP, therefore increased the spatial vulnerability to reentry arrhythmias. (iii) The T618I mutation caused a series of remodeling effects on the restitution properties, including the flattened APD restitution curve and the reduced rate adaption of the QT interval. (iv) The T618I mutation stabilized the reentrant spiral waves, which was evidenced by the increased rotation frequency and the small meandering size of spiral wave tips. Besides, 2D and 3D simulations consistently showed that the T618I mutation favored the sustenance of reentry arrhythmias on realistic tissues of limited size. (v) Quinidine prolonged APD and ERP in all cell types, and restored the QT interval in the mutation condition. (vi) Quinidine reversed the flattened APD restitution curve under the mutation condition and rescued the rate adaption of the QT interval as well. (vii) Quinidine decreased the spatial vulnerability to reentry arrhythmias, reduced the rotation frequency of spiral waves, and also successfully prevented the sustained spiral/scroll waves in realistic slice and organ models of SQTS. (viii) Clinically relevant doses of sotalol failed to inhibit mutant *I*_Kr_, but the failure reasons were different between N588K and T618I.

### Relevance to previous experimental studies

The existing understanding of SQTS is mainly derived from three experimental models, namely drug-induced models, induced pluripotent stem cell (iPSC)-based models, and transgenic animal models. First, drugs like pinacidil and specific *I*_Kr_ agonists can act on outward potassium currents and lead to reduction of APD and QT interval, therefore they were used in early studies to mimic SQTS^19–22^. Although drug-induced SQTS models have allowed for a better understanding of the SQTS mechanism, the drug may also activate ion channels not implicated in SQTS, or affect *I*_Kr_ biophysics in a different way from the mutation induced functional remodeling^23^. Therefore, experimental SQTS models based on induced pluripotent stem cell cardiomyocytes (iPSC-CMs) and transgenic animals have emerged as new tools for investigating SQTS. Guo et al.^24^ generated iPSC-CMs with *KCNH2* T618I from skin fibroblasts and demonstrated that iPSC-CMs could recapitulate the increased current density of *I*_Kr_ and abnormal AP phenotype featured by shortened APD. Similar techniques were also adopted for generating iPSC-CMs of another SQT1 subtype carrying *KCNH2* N588K^25^. El-Battrawy et al.^26^ established a cellular model of SQTS using hiPSC-CMs, and most of our simulation results were consistent with their observations. Specifically, they showed that most of the AP biomarkers including resting potential (RP), action potential amplitude (APA), maximal upstroke velocity (*V*_max_) did not exhibit statistically significant differences between WT and SQTS groups. APD_50_ and APD_90_ were the only two parameters that showed differences. In our simulations, take EPI cells as examples, the RP changed from −85.47 mV (WT) to −85.62 mV (mutation), the APA changed from 127.01 mV (WT) to 127.15 mV (mutation), and the *V*_max_ changed from 387.23 mV (WT) to 390.81 mV (mutation). The insensible changes of the above three parameters are consistent with El-Battrawy et al.’s observations. On the other hand, our simulations reproduced the significantly decreased APD_50_ and APD_90_ in experiments. Specifically, APD_90_ dropped from 308.94 ms (WT) to 235.16 ms (mutation), and APD_50_ decreased from 276.16 ms (WT) to 203.12 ms (mutation). Therefore, the constructed model in this study is able to recapitulate the phenotype of SQT1 at the cellular level. The simulation results support El-Battrawy et al.’s observations.

El-Battrawy et al. reported an interesting observation that the intracellular Ca^2+^ level was increased in the SQTS group. Arrhythmia events (manifested as early or delayed afterdepolarization-like Ca^2+^ transients) were also frequently observed. To explore the underlying mechanisms, we conducted additional experiments and recorded the changes in Ca^2+^ transients under mutation conditions. However, our simulation showed that the intracellular Ca^2+^ concentration was decreased and no EAD-like Ca^2+^ activities were observed (see Supplementary Fig. 1). Identical observations were observed in another two cell models (i.e., ORd and ToR-ORd) so that the factor of model-dependency can be excluded. In our simulations, the decreased Ca^2+^ concentration was attributed to the action potential abbreviation-induced *I*_CaL_ decreasing, which in turn affected the intracellular Ca^2+^ released from the sarcoplasmic reticulum. Noted that the decreased Ca^2+^ was also observed in another simulation study^27^. Therefore, the underlying mechanisms for the increased Ca^2+^ in SQTS were currently unknown. Although the increased Ca^2+^ was not reproduced in simulations, the observed afterdepolarization activities in^26^ are of great significance for understanding arrhythmogenesis in SQTS patients. Current studies have revealed the increased vulnerability to arrhythmias (substrates for arrhythmias) in hearts with SQTS mutations, while few is known about the actual “triggers”. Therefore, the EAD-like Ca^2+^ activities warrant further investigations by means of wet experiments.

In terms of the pharmacotherapy, Zhao et al.^28^ tested the effects of six clinical drugs on hiPSC-CMs. Among six tested drugs, three of them, namely ivabradine, ajmaline, and mexiletine, were observed to prolong APD and reduce arrhythmic events. In ref. ^27^, Jæger et al. computed the optimal drug combinations for SQT1 based on a suite of computational models including hiPSC-CMs, rabbit ventricular myocytes, and adult human ventricular myocytes. They proposed a reliable in silico approach for assessing the combined efficacy of multiple drugs, and they suggested that simultaneous induction of *I*_NaL_ and blockade of *I*_Kr_ could be a promising strategy for SQT1.

Despite the great achievements of hiPSC-CMs, cell models cannot reflect those dynamic behaviors at the tissue level. In view of this, Shinnawi et al.^29^ established a iPSC-based 2D model named “hiPSC-CCSs” (i.e., human iPSC-derived cardiac cell sheets) to study the tissue-level SQTS phenotype. Finally, transgenic SQTS animals are the third experimental measure to investigate the mechanisms underlying SQTS. Compared with iPSC-based myocytes and tissue models, transgenic SQTS animals can mimic the human disease phenotype on all levels (from ion current to whole-heart and in vivo levels). For example, Odening et al.^23^ raised transgenic SQT1 rabbits carrying *KCNH2* N588K by which the whole-heart and in vivo phenotypes of SQT1 such as the increased QT-dispersion in 12-lead ECG and the lack of QT interval adaption to heart rates were observed.

The virtual heart model in this study provided another reliable tool for investigating the mechanisms of SQTS and evaluating the pharmacotherapeutic effects of antiarrhythmic drugs. Different from some current data-driven models^30^, the presented model recapitulated many clinically or experimentally observed important phenotypes of SQT1 as well as the drug effects at multiple levels, thus facilitating our understanding of the mechanism underlying SQTS. Specifically, at the cellular level, the cell model reproduced the abbreviated APD and ERP widely reported in previous experimental research^22,31^. In particular, the APD restitution-plots (Fig. 2A) recapitulated the reduced rate adaption of APD, which was a critical cellular level phenotype of SQTS^23,29^. For the phenotype at the tissue level, Shinnawi et al. generated iPSC-based tissue of SQTS and demonstrated the marked shortening of wavelength without affecting CV, the increased susceptibility for reentry arrhythmias, and the increased rotor frequency^29^. In line with these observations, our tissue model also produced an unaffected CV (at normal heart rates) and shortened wavelength. Increased spatial vulnerability and rotor frequency were also observed in our simulation. Third, the model successfully recapitulated the impaired QT interval adaption to heart rates. The reduced rate adaption of QT particularly at fast heart rates is an important ECG phenotype of SQTS^23,32^ and has been utilized as a diagnostic tool in clinical practice^12,33^. Finally, the drug quinidine showed a strong ability to rescue abnormal SQTS phenotypes at different levels. Available experimental and clinical studies have reached a consensus on the efficacy of ranolazine in saving SQTS hearts from arrhythmias^33–35^. Our simulation results regarding the effects of quinidine were consistent with these studies. As summarized in the Main Finding section, simulations demonstrated that quinidine prolonged APD and restored QT interval at cellular and organ levels, respectively. The decreased inducibility of arrhythmias and reduced rotor frequency predicted by the model after the “administration” of quinidine were also observed in the iPSC-based tissue model^29^.

This study provides theoretical insights into the difference of drug efficacy between quinidine and sotalol. Based on evaluation results of five drugs, McPate et al. summarized that SQT1 might be more responsive to those hERG blockers that do not depend strongly on inactivation for their potency^36^. Particularly, they suggested that quinidine’s ability to block N588K-hERG at therapeutic concentrations might derive from its comparative insensitivity to attenuation of hERG inactivation. In accordance with McPate et al.’s findings, our simulation results revealed that quinidine’s ability to block hERG depended more on the Open state and was less sensitive to the change of the Inactivation state compared with sotalol. This can be reflected in a quantitative way by introducing the ratio *k*_*A*_/*k*_*I*_, where *k*_*A*_ and *k*_*I*_ are transition rates from Open (*O*) to Drug-bound Open (*OD*), and from Inactivation (*I*) to Drug-bound Inactivation (*ID*), respectively. The ratio is able to demonstrate the extent to which the blocking capacity of a drug depends on the Open state (alternatively, not depend on the Inactivation state). The *k*_*A*_*/k*_*I*_ are separately 23.8 and 1.50 for quinidine and sotalol (see Eqs. (66), (68), (85), (87) in Methods), which clearly shows the discrepancy of sensitivity to inactivation between these two drugs. The higher *k*_*A*_*/k*_*I*_ value of quinidine suggests that it is less sensitive to inactivation than sotalol, which is consistent with McPate’s inference.

Of note, there are some simulation results that do not agree with previously reported experimental findings. Specifically, experiments based on iPSC-derived cell sheets demonstrated that there was no obvious difference between the CVs measured under conditions of mutation and quinidine administration^29^. In contrast, our simulations showed that the CVs diverge significantly under these two conditions (Fig. 9B). In our model, the drug effects of quinidine were simulated by incorporating its dose-dependent influences on multiple ion channels, among which the suppression of *I*_Na_ would cause a direct impairment on the conduction of excitations. Quinidine is a widely acknowledged class Ia antiarrhythmic agent, and the unchanged CV in experiments might be due to the considerably slow velocity of only around 2.5 cm/s in the iPSC-derived cell sheet (for comparison, the CV is about 70 cm/s in physiological conditions and in our simulations). Another inconsistence that may arise from the slow conduction velocity was the meandering activity of rotor tips. Shinnawi et al.^29^ showed that the induced spiral wave in the wild-type cell sheet meandered greatly, while the spiral wave in the mutation condition became more stable with decreased meandering distance of the rotor tip. Although the model recapitulated the stable spiral wave and increased rotor frequency, it failed to reproduce the meandering behavior of the spiral wave tip under the wild-type condition.

As far as we are concerned, this study is the first one to simulate failed drugs using identical mathematical equations. For example, Luo et al.^16^ found that E-4031 and disopyramide failed to exert noticeable effects on APD; however, the interaction of drugs with *I*_Kr_ was modeled based on simple Hill functions and different parameters (e.g., IC_50_) were used for WT and mutation conditions. In contrast, we modeled the effects of sotalol by adding exactly the same two states (along with identical association/dissociation rates, see Eqs. (85)–(88), (98)–(101) in Methods) to the original 5-state Markov chain model. Another simulation study by Whittaker et al.^13^ discussed the potential antiarrhythmic effects of disopyramide and quinidine on SQT1. However, the significantly different effects of those failed class III drugs were neither simulated nor analyzed. In our study, the two pharmacological states were able to account for various conditions. It could not only reproduce the significant *I*_Kr_-blocking effects of class III drugs (i.e., sotalol) under the WT condition, but also accurately replicate the decreased efficacy under T618I and N588K mutations.

### Mutation-dependent failure reasons of sotalol in SQT1 patients

Sotalol is a typical class III antiarrhythmic drug known to block *I*_Kr_, but clinical studies have proved it to be a failed drug therapy for SQT1 patients^17,25^. In addition, experimental investigations demonstrated that the loss of drug effects were not the same among different SQT1 mutations^11^. Our simulations showed that, for N588K, the inhibition ability of sotalol on *I*_Kr_ was significantly diminished and even high doses of sotalol (500 μmol) did not exert enough inhibitory effects over the physiological membrane potential range. McPate et al. predicted that for drugs strongly depend on inactivation, the gap between potency of inhibition of N588K and WT *I*_Kr_ might be smaller at more positive voltages. This assumption is also proved using our models. To be more specific, when clamped at 20 mV, 100 μmol sotalol blocked *I*_Kr_ by 49.4% and 7.5% under WT and N588K conditions, respectively, and such gap was significantly restored at more positive voltages (>60 mV, see Supplementary Fig. 18). Despite the restored inhibiting ability, the voltages that sotalol acted inhibitory effects were out of physiological action potential range (about −90 to 20 mV).

Compared with N588K, the drug efficacy was less diminished in the T618I condition. For example, 500 μmol sotalol was still able to inhibit *I*_Kr_ by 63.8% at +20 mV under the T618I condition, which is in accordance with Sun et al.’s experimental observations that 500 μmol sotalol achieved comparable inhibitory effect to 5 μmol quinidine^11^. However, the required dose (i.e., 500 μmol) is beyond the plasma concentrations (88.4–265.2 μmol) during long-term oral therapy^17^.

Taken together, this study reveals mutation-dependent failure reasons of sotalol. Although the failure of sotalol can be generally explained as its high sensitivity to the Inactivation state, the detailed processes are not exactly the same for N588K and T618I. Through a review of past literature, there are few studies building Markov models for this drug, and even fewer in silico research focused on the reason why sotalol failed in SQT1 patients. In this regard, Brennan et al.^37^ proposed a 10-state Markov model of sotalol where two parallel state groups (i.e., each group had five states: C1, C2, C3, I, O) were included. The model was validated on the inhibition time course of sotalol (Fig. 3 in ref. ^38^), and again, the significantly different effects of sotalol under control and T618I conditions were not simulated or discussed in their study. Besides, the model proposed by Brennan et al. was based on “guarded-receptor theory”^38^, i.e., the drug molecule binds with only the open state. However, previous studies have shown that the binding of drugs with the inactivation state is also necessary for modeling drug actions^13,39^. Our study also supports the necessity of adding the drug-bound inactivation (“ID”) state, as the model could replicate the altered drug efficacy of sotalol only when both OD and ID were incorporated.

### Limitations

Previous studies have suggested that *I*_Kr_ is not the only changed ion current in SQT1 patients. Specifically, Guo et al.^24^ generated iPSC-CMs from a SQT patient carrying *KCNH2* T618I mutation, where the mRNA-level expression of *SCN5A*, KCNQ1, and CACNA1C were all significantly elevated. In line with these increased mRNA-level expressions, the corresponding currents including *I*_Na_, *I*_Ks_, and *I*_CaL_ were all significantly increased. Such remodeling effects were observed even after the correction of T618I mutation in SQTS iPSC-CMs. In addition to T618I, electrical remodeling was also observed in another common variant of SQT1, *KCNH2* N588K. Cellular experiments based on transgenic rabbits that carried *KCNH2* N588K reported that *I*_K1_ was decreased while *I*_Ks_ was increased in SQT1 myocytes compared with WT^23^. The above observations together indicate that the consequences of mutations to a specific gene are not limited to its corresponding ionic current, but may also result in remodeling of multiple ion channels. Such remodeling effects are not considered in the present study but warrant further investigations in the future.

The monodomain model, rather than the bidomain model, was adopted in this study. According to previous research, the monodomain model is significantly superior to the bidomain model in terms of computational cost, and in some cases the monodomain model can be more than 10 times faster for same problems^40^. On the other hand, previous literatures that discussed the difference in accuracy between bidomain and monodomain models reported that these two models produced almost identical results except in some cases where stimulus were injected into the extracellular space (e.g., defibrillation). Specifically, Potse et al.^41^ investigated the impact of the monodomain assumption on simulated propagation by comparing its results with a bidomain model. They concluded that differences between the two models were extremely small, and repeated experiments with simulated ischemia or sodium conductivity reduced by 90% arrived at the same conclusion. Bourgault et al.^42^ compared monodomain and bidomain models in terms of the activation time, and they performed simulations on both idealized geometry and realistic 2D slice. They concluded that the discrepancy between the two models was of order 1% or even below, and it was smaller than the discretization error resulting from commonly used mesh size. A good review by Clayton et al.^43^ summarized that if there is no injection of current into the extracellular space, AP propagation provided by mono- and bidomain models are close to each other even under the condition of unequal anisotropy ratio. In our case, there was no stimulus applied to the extracellular space, and therefore we used the monodomain model for simulations.

It should also be noted that the mechanical contraction was not considered in this study. All the ventricular models including the idealized models and the realistic 2D slice and the 3D ventricle were assumed to be stationary. However, the ventricle was known to contract during its repolarization phase, and the geometry would be altered. The altered geometry, along with the mechano-electric feedback, may further influence the T-wave configuration and the electrophysiological behavior^44,45^. Therefore, the potential roles of mechanical contraction and mechano-electric feedback warrant further investigations.

## Conclusion

This study concluded that the arrhythmogenesis of the *KCNH2* T618I mutation arises from a series of electrophysiological alterations caused by the enhanced *I*_Kr_. The T618I mutation shortened the critical length for initiating reentrant excitation waves by abbreviating the ventricular ERP, therefore facilitated the genesis and maintenance of reentry arrhythmias. The antiarrhythmic drug quinidine was suggested to be an effective treatment as it prolonged the cellular ERP, restored the abbreviated QT interval, and also removed substrates for reentry arrhythmias in SQTS patients by not only prolonging the critical length for initiating spiral waves but also preventing the persistence of those evoked ones. These observations were highly consistent when simulations were performed using three apparently different cell models, suggesting the robustness of the developed Markov model and the model-independence of the reported findings. In summary, the study establishes a causal linkage between the genetic mutation and the organ-level ventricular arrhythmias and clinical ECG observations, and the developed Markov *I*_Kr_ model and all the upper level models ranging from cells to organs together constitute a reliable platform for not only the investigation of the underlying mechanisms of SQT1, but also the screening of effective antiarrhythmic drugs for SQT1 patients.

## Methods

### Development of Markov chain models for *I*_Kr_ in wild-type and *KCNH2* T618I mutation conditions

The Hodgkin–Huxley model (HH model) assumes that the channel gates are independent; however, experiments have shown that activation and inactivation processes are typically dependent on each other. As a result, explicit representations of ion channel states are necessary, as in the case of Markov chain models. Jæger et al. mentioned that the Markov model is able to give a more realistic representation of both the effect of mutations and the effect of drugs^46^, and the superiority of the Markov model was also explicitly demonstrated in other simulation studies^47^. Therefore, the Markov chain model of *I*_Kr_ developed in this study is more accurate than the HH-based *I*_Kr_ formulations. A general introduction to the use of Markov models can be found in ref. ^48^.

The Markov chain model for *I*_Kr_ in this study was based on a previous *I*_Kr_ model by Clancy and Rudy^49^, as illustrated in Fig. 1B. The model contained five states, including an open state (O), an inactivated state (I), and three closed states (C1, C2, C3). The model parameters were optimized by minimizing the least-squared difference between the model-generated current–voltage (I–V) curve and the experimentally recorded I–V data. The optimizing process was performed using the LMFIT package that implemented in Python. Model equations and the fitted transition rates are listed in Eqs. (1)–(25).

#### *I*_Kr_ Markov chain model formulations

1 $$I_{{{{\mathrm{Kr}}}}} = G_{{{{\mathrm{Kr}}}}} \times O \times (V_{{{\mathrm{m}}}} - E_{{{\mathrm{K}}}})$$

2 $$E_{{{\mathrm{K}}}} = \frac{{RT}}{F}\log \frac{{{{{\mathrm{[K}}}}^ + {{{\mathrm{]}}}}_{{{\mathrm{o}}}}}}{{{{{\mathrm{[K}}}}^ + {{{\mathrm{]}}}}_{{{\mathrm{i}}}}}}$$

3 $$C_1 = \beta \cdot C_2 - \alpha \cdot C_1$$

4 $$C_2 = \alpha \cdot C_1 - \beta \cdot C_2 + \beta _1 \cdot C_3 - \alpha _1 \cdot C_2$$

5 $$C_3 = \alpha _1 \cdot C_2 - \beta _1 \cdot C_3 - 2 \cdot \alpha _2 \cdot C_3 + \mu \cdot I + \beta _2 \cdot O$$

6 $$I = \alpha _2 \cdot C_3 - \mu \cdot I + \alpha _i \cdot O - \beta _i \cdot I$$

7 $$O = 1 - (C_1 + C_2 + C_3 + I)$$

#### Transition rates for the wild-type channel (ms^−1^)

8 $$\alpha _1 = 2.172$$

9 $$\beta _1 = 1.077$$

10 $$\alpha _2 = 0.00655 \times e^{0.027735765 \times (V - 61)}$$

11 $$\beta _2 = 0.001908205 \times e^{ - 0.0148902 \times V}$$

12 $$\alpha _i = 0.04829 \times e^{ - 0.039984 \times V} \times \left( {\frac{{4.5}}{{[{{{\mathrm{K}}}}^ + ]_{{{\mathrm{o}}}}}}} \right)$$

13 $$\beta _i = 0.2624 \times e^{0.000942 \times V} \times \left( {\frac{{4.5}}{{[{{{\mathrm{K}}}}^ + ]_{{{\mathrm{o}}}}}}} \right)^{0.3}$$

14 $$\alpha = 0.00555 \times e^{0.05547153 \times (V - 36)}$$

15 $$\beta = 0.002357 \times e^{ - 0.036588 \times V}$$

16 $$\mu = \frac{{\alpha _i\beta _2}}{{\beta _i}}$$

#### Transition rates for the T618I mutant channel (ms^−1^)

17 $$\alpha _1 = 2.172$$

18 $$\beta _1 = 1.077$$

19 $$\alpha _2 = 0.00655 \times e^{0.027735765 \times (V - 57)}$$

20 $$\beta _2 = 0.001908205 \times e^{ - 0.0148902 \times V}$$

21 $$\alpha _i = 0.24145 \times e^{ - 0.039984 \times (V + 4)} \times \left( {\frac{{4.5}}{{[{{{\mathrm{K}}}}^ + ]_{{{\mathrm{o}}}}}}} \right)$$

22 $$\beta _i = 0.05248 \times e^{0.000942 \times V} \times \left( {\frac{{4.5}}{{[{{{\mathrm{K}}}}^ + ]_{{{\mathrm{o}}}}}}} \right)^{0.3}$$

23 $$\alpha = 0.00555 \times e^{0.05547153 \times (V - 33)}$$

24 $$\beta = 0.002357 \times e^{ - 0.036588 \times V}$$

25 $$\mu = \frac{{\alpha _i\beta _2}}{{\beta _i}}$$

#### Transition rates for the N588K mutant channel (ms^−1^)

26 $$\alpha _1 = 2.172$$

27 $$\beta _1 = 1.077$$

28 $$\alpha _2 = 0.00655 \times e^{0.027735765 \times (V - 57)}$$

29 $$\beta _2 = 0.005724615 \times e^{ - 0.0148902 \times V}$$

30 $$\alpha _i = 0.38632 \times e^{ - 0.039984 \times (V + 4)} \times \left( {\frac{{4.5}}{{[{{{\mathrm{K}}}}^ + ]_{{{\mathrm{o}}}}}}} \right)$$

31 $$\beta _i = 0.0328 \times e^{0.000942 \times V} \times \left( {\frac{{4.5}}{{[{{{\mathrm{K}}}}^ + ]_{{{\mathrm{o}}}}}}} \right)^{0.3}$$

32 $$\alpha = 0.00555 \times e^{0.05547153 \times (V - 33)}$$

33 $$\beta = 0.002357 \times e^{ - 0.036588 \times V}$$

34 $$\mu = \frac{{\alpha _i\beta _2}}{{\beta _i}}$$

### Modeling multi-channel effects of quinidine

Quinidine was known to affect multiple ionic currents including *I*_Kr_^11,50^, *I*_Ks_^51^, *I*_K1_^52^, *I*_to_^52^, *I*_CaL_^53^, *I*_Na_^54,55^, and *I*_NaL_^56^. In this study, we incorporated all these reported ionic currents into their corresponding ion channel models. Specifically, for the *I*_Kr_, two additional states related to drugs were added to the Markov chain model as in^13^ (Fig. 7B). Model equations and the fitted transition rates are listed in Eqs. (35)–(69).

#### I_Kr_ Markov chain model formulations

35 $$I_{{{{\mathrm{Kr}}}}} = G_{{{{\mathrm{Kr}}}}} \times O \times (V_{{{\mathrm{m}}}} - E_{{{\mathrm{K}}}})$$

36 $$E_{{{\mathrm{K}}}} = \frac{{RT}}{F}\log \frac{{{{{\mathrm{[K}}}}^ + {{{\mathrm{]}}}}_{{{\mathrm{o}}}}}}{{{{{\mathrm{[K}}}}^ + {{{\mathrm{]}}}}_{{{\mathrm{i}}}}}}$$

37 $$C_1 = \beta \cdot C_2 - \alpha \cdot C_1$$

38 $$C_2 = \alpha \cdot C_1 - \beta \cdot C_2 + \beta _1 \cdot C_3 - \alpha _1 \cdot C_2$$

39 $$C_3 = \alpha _1 \cdot C_2 - \beta _1 \cdot C_3 - 2 \cdot \alpha _2 \cdot C_3 + \mu \cdot I + \beta _2 \cdot O$$

40 $$I = \alpha _2 \cdot C_3 - \mu \cdot I + \alpha _i \cdot O - \beta _i \cdot I + I_l \cdot ID - I \cdot k_l[D]$$

41 $$O = \alpha _2 \cdot C_3 - \beta _2 \cdot O + \alpha _i \cdot I - \beta _i \cdot O + I_A \cdot OD - k_A[D] \cdot O$$

42 $$ID = k_l[D] \cdot I - I_l \cdot ID$$

43 $$OD = 1 - (C_1 + C_2 + C_3 + I + O + ID)$$

#### Transition rates for the T618I mutant channel after the application of quinidine (ms^−1^)

44 $$\alpha _1 = 2.172$$

45 $$\beta _1 = 1.077$$

46 $$\alpha _2 = 0.00655 \times e^{0.027735765 \times (V - 57)}$$

47 $$\beta _2 = 0.001908205 \times e^{ - 0.0148902 \times V}$$

48 $$\alpha _i = 0.24145 \times e^{ - 0.039984 \times (V + 4)} \times \left( {\frac{{4.5}}{{[{{{\mathrm{K}}}}^ + ]_{{{\mathrm{o}}}}}}} \right)$$

49 $$\beta _i = 0.05248 \times e^{0.000942 \times V} \times \left( {\frac{{4.5}}{{[{{{\mathrm{K}}}}^ + ]_{{{\mathrm{o}}}}}}} \right)^{0.3}$$

50 $$\alpha = 0.00555 \times e^{0.05547153 \times (V - 33)}$$

51 $$\beta = 0.002357 \times e^{ - 0.036588 \times V}$$

52 $$\mu = \frac{{\alpha _i\beta _2}}{{\beta _i}}$$

53 $$k_I = 1.91 \times 10^{ - 4.0}$$

54 $$l_I = 1.29 \times 10^{ - 5.0}$$

55 $$k_A = 1.44 \times 10^{ - 2.5}$$

56 $$l_A = 4.37 \times 10^{ - 3.0}$$

#### Transition rates for the N588K mutant channel after the application of quinidine (ms^−1^)

57 $$\alpha _1 = 2.172$$

58 $$\beta _1 = 1.077$$

59 $$\alpha _2 = 0.00655 \times e^{0.027735765 \times (V - 57)}$$

60 $$\beta _2 = 0.005724615 \times e^{ - 0.0148902 \times V}$$

61 $$\alpha _i = 0.38632 \times e^{ - 0.039984 \times (V + 4)} \times \left( {\frac{{4.5}}{{[{{{\mathrm{K}}}}^ + ]_{{{\mathrm{o}}}}}}} \right)$$

62 $$\beta _i = 0.0328 \times e^{0.000942 \times V} \times \left( {\frac{{4.5}}{{[{{{\mathrm{K}}}}^ + ]_{{{\mathrm{o}}}}}}} \right)^{0.3}$$

63 $$\alpha = 0.00555 \times e^{0.05547153 \times (V - 33)}$$

64 $$\beta = 0.002357 \times e^{ - 0.036588 \times V}$$

65 $$\mu = \frac{{\alpha _i\beta _2}}{{\beta _i}}$$

66 $$k_I = 1.91 \times 10^{ - 4.0}$$

67 $$l_I = 1.29 \times 10^{ - 5.0}$$

68 $$k_A = 1.44 \times 10^{ - 2.5}$$

69 $$l_A = 4.37 \times 10^{ - 3.0}$$

#### The effects of quinidine on other channel currents

For the other currents, the dose-dependent drug effects were modeled based on the “pore block” theory^57^. The fitted equations are as Eqs. (70)–(75).

70 $$k_{IKs} = \frac{{1.0}}{{1.0 + \left( {\frac{{43.01}}{{[QUIN]}}} \right)^{1.88}}}$$

71 $$k_{IK1} = \frac{{1.0}}{{1.0 + \left( {\frac{{99.74}}{{[QUIN]}}} \right)^{0.2765}}}$$

72 $$k_{Ito} = \frac{{1.0}}{{1.0 + \left( {\frac{{20.75}}{{[QUIN]}}} \right)^{0.6671}}}$$

73 $$k_{INa} = \frac{{1.0}}{{1.0 + \left( {\frac{{14.60}}{{[QUIN]}}} \right)^{1.22}}}$$

74 $$k_{INaL} = \frac{{1.0}}{{1.0 + \left( {\frac{{10.41}}{{[QUIN]}}} \right)^{0.8320}}}$$

75 $$k_{ICaL} = \frac{{1.0}}{{1.0 + \left( {\frac{{15.10}}{{[QUIN]}}} \right)^{1.1369}}}$$

“*k*_current_” in above equations is the blocking factor that represents the reduction ratio of the maximum conductance of the targeted ion channel. “[QUIN]” represents the concentration of quinidine in μmol. The fitting results are illustrated in Supplementary Fig. 19. Experimental data sources^11,50–56^ are indicated in the figure caption.

### Modeling the effect of sotalol on *I*_Kr_

The Markov model of sotalol on *I*_Kr_ was identical to that of quinidine (Fig. 7B and Eqs. (35)–(43)), except that the transition rates were adjusted to recapitulate experimental observations. The transition rates under T618I and N588K conditions are listed in subsections “Transition rates for the T618I mutant channel after the application of sotalol (ms^−1^)” and “Transition rates for the N588K mutant channel after the application of sotalol (ms^−1^)”, respectively.

#### Transition rates for the T618I mutant channel after the application of sotalol (ms^−1^)

76 $$\alpha _1 = 2.172$$

77 $$\beta _1 = 1.077$$

78 $$\alpha _2 = 0.00655 \times e^{0.027735765 \times (V - 57)}$$

79 $$\beta _2 = 0.001908205 \times e^{ - 0.0148902 \times V}$$

80 $$\alpha _i = 0.24145 \times e^{ - 0.039984 \times (V\, + \,4)} \times \left( {\frac{{4.5}}{{[{{{\mathrm{K}}}}^ + ]_{{{\mathrm{o}}}}}}} \right)$$

81 $$\beta _i = 0.05248 \times e^{0.000942 \times V} \times \left( {\frac{{4.5}}{{[{{{\mathrm{K}}}}^ + ]_{{{\mathrm{o}}}}}}} \right)^{0.3}$$

82 $$\alpha = 0.00555 \times e^{0.05547153 \times (V - 33)}$$

83 $$\beta = 0.002357 \times e^{ - 0.036588 \times V}$$

84 $$\mu = \frac{{\alpha _i\beta _2}}{{\beta _i}}$$

85 $$k_I = 3.02715 \times 10^{ - 6.0}$$

86 $$l_I = 1.29 \times 10^{ - 5.0}$$

87 $$k_A = 4.55368 \times 10^{ - 6.0}$$

88 $$l_A = 4.37 \times 10^{ - 3.0}$$

#### Transition rates for the N588K mutant channel after the application of sotalol (ms^−1^)

89 $$\alpha _1 = 2.172$$

90 $$\beta _1 = 1.077$$

91 $$\alpha _2 = 0.00655 \times e^{0.027735765 \times (V - 57)}$$

92 $$\beta _2 = 0.005724615 \times e^{ - 0.0148902 \times V}$$

93 $$\alpha _i = 0.38632 \times e^{ - 0.039984 \times (V + 4)} \times \left( {\frac{{4.5}}{{[{{{\mathrm{K}}}}^ + ]_{{{\mathrm{o}}}}}}} \right)$$

94 $$\beta _i = 0.0328 \times e^{0.000942 \times V} \times \left( {\frac{{4.5}}{{[{{{\mathrm{K}}}}^ + ]_{{{\mathrm{o}}}}}}} \right)^{0.3}$$

95 $$\alpha = 0.00555 \times e^{0.05547153 \times (V - 33)}$$

96 $$\beta = 0.002357 \times e^{ - 0.036588 \times V}$$

97 $$\mu = \frac{{\alpha _i\beta _2}}{{\beta _i}}$$

98 $$k_I = 3.02715 \times 10^{ - 6.0}$$

99 $$l_I = 1.29 \times 10^{ - 5.0}$$

100 $$k_A = 4.55368 \times 10^{ - 6.0}$$

101 $$l_A = 4.37 \times 10^{ - 3.0}$$

### Single cell simulation

The human ventricular myocyte model developed by Ten Tusscher et al. (TNNP06 model)^58^ was used in this study. The electrophysiological behavior at the cellular level was described as:

102 $$\frac{{\partial V_{{{\mathrm{m}}}}}}{{\partial t}} = - \frac{{I_{{{{\mathrm{ion}}}}} + I_{{{{\mathrm{stim}}}}}}}{{C_{{{\mathrm{m}}}}}}$$

where *V*_m_ is the membrane potential, *I*_ion_ and *I*_stim_ are separately the total ionic current and the stimulating current, *C*_m_ is the membrane capacitance.

Next, we replaced the original *I*_Kr_ in the TNNP06 model by the aforementioned Markov chain model. Besides, the heterogeneity of *I*_Kr_ in different cell types was considered in this study, and the ratio of the maximum conductance of *I*_Kr_ in EPI:MCELL:ENDO was set to 1.6:1.0:1.0 based on previous experimental measurements^59^. The modified TNNP06 model was paced to achieve its steady state by fifty supra-threshold stimuli (52 pA/pF, 1 ms) in a frequency of 1.25 Hz, which corresponds to a normal heart rate of 75 beats/min. The last AP was recorded for measuring its parameters, e.g., APD, overshoot, etc.

The effective refractory period (ERP) was measured by a standard S1–S2 protocol. Specifically, fifty S1 stimuli were applied at 1.25 Hz before a premature stimulus (S2) was applied. The S2 had the same amplitude and duration as S1, and it would not be able to evoke a new action potential if being applied within the refractory period after the last S1. The protocol was conducted iteratively by gradually reducing the S1–S2 interval, and the ERP was measured as the smallest diastolic interval when the S2 was able to evoke an AP that reached 80% of the overshoot of the preceding AP^60^.

### Multi-scale virtual human ventricle

The propagation of excitation waves was described using the monodomain equation^43^,

103 $$\frac{{\partial V_{{{\mathrm{m}}}}}}{{\partial t}} = \nabla \cdot {{{\bf{D}}}}(\nabla V_{{{\mathrm{m}}}}) - \frac{{I_{{{{\mathrm{ion}}}}} + I_{{{{\mathrm{stim}}}}}}}{{C_{{{\mathrm{m}}}}}}$$

where **D** is the diffusion coefficient tensor for describing the intercellular electrical coupling via gap junctions.

For one-dimensional (1D) simulations, a 15 mm long transmural 1D strand of 100 nodes with spacing 0.15 mm was constructed. The strand length was consistent with the normal range of human transmural ventricle width (4–14 mm) in previous studies^61,62^. The ratio of ENDO:MCELL:EPI was set to 37:26:37 as in previous study^14^. The diffusion coefficient was set to 0.154 mm^2^/ms to get a conduction velocity of 71.9 cm/s, which was very close to the 70 cm/s that recorded in the human myocardium^63^.

The 1D strand was expanded in the *y* direction to form a 15 × 60 mm^2^ two-dimensional (2D) tissue sheet. Isotropic cell-to-cell coupling was assumed in the idealized model, and the isotropic coefficient was kept the same as in the 1D strand. For the realistic 2D ventricle tissue slice, the **D** was anisotropic and the coefficients along and perpendicular to the fiber orientation were set to 0.154 and 0.0385 mm^2^/ms, respectively. For the realistic three-dimensional (3D) bi-ventricle geometry, the anisotropic coefficients were the same as in the 2D realistic tissue slice. The anatomical geometries of realistic 2D ventricular slice and 3D ventricle were reconstructed from DT-MRI^64,65^.

### Generation of pseudo-ECG

The pseudo-ECG was generated using Eqs. (104) and (105)^66^:

104 $$\Phi (x^\prime ,y^\prime ,z^\prime ) = \frac{{a^2\sigma _i}}{{4\sigma _e}}{\int} {( - \nabla V_m) \cdot \left[ {\nabla \frac{1}{r}} \right]d\Omega }$$

105 $$r = \left[ {(x - x^\prime )^2 + (y - y^\prime )^2 + (z - z^\prime )^2} \right]^{\frac{1}{2}}$$

where $\Phi$ is a unipolar potential generated by the tissue, *r* is the distance between a source point and the virtual electrode, $\sigma _{\it{i}}$ and $\sigma _{\it{e}}$ stand for intracellular and extracellular conductivities, respectively, and ${\int} {}$ is the domain of integration.

The corrected QT interval (QTc) was obtained from the simulated ECG. It was measured as the interval from the earliest onset of the depolarization wave to the end of the T-wave. The measured QT was corrected for heart rate using the Bazett equation:

106 $$QTc = QT/\sqrt {RR}$$

where RR is the interval between two consecutive R waves.

### APD and CV restitution curves and rate adaption curve of QT interval

Two protocols namely the standard S1–S2 protocol and the dynamic protocol were adopted to determine the APD and CV restitution curves. More concretely, for the APD restitution curve (APDR), a series of stimuli (S1) at a frequency of 1.25 Hz were applied to the single cell model until it reached its steady states. A premature stimulus (S2) was then delivered at some diastolic interval (DI) after the last AP of S1. The APDR could be obtained by plotting the APD of the S2-induced AP against DI. In addition to the APD-DI restitution curve generated by using the S1–S2 protocol mentioned above, the APD-PCL was also determined by the dynamic protocol. The cell model was paced 50 times with an initial cycle length of 1000 ms, and the APD of the last AP was recorded, after which the cycle length was decreased to measure the new APD. The APDR was determined by plotting APD against PCL (pacing cycle length).

For the CV restitution curve, S1 stimuli of 1.25 Hz were applied to a 15-mm long tissue strand. Then S2 was applied at a DI after the last S1 excitation wave. The CV of the S2-induced excitation wave was measured and plotted against DI. In addition, similar to the APD-PCL curve, CV-PCL restitution curve was obtained using the dynamic protocol.

For the rate adaption curve of QT interval, similar dynamic protocol was used. The 1D tissue strand was paced 10 times under each PCL to generate a “steady-state” pseudo-ECG where the QT was measured using the aforementioned method.

### Measurement of the vulnerable window to the unidirectional conduction block

The vulnerable window (VW) is a certain period when a premature stimulus applied to the refractory tail of the previous excitation wave will result in a unidirectional conduction block. The VW was measured using the S1–S2 protocol in the 1D strand model. Specifically, the S1 stimulus was applied to the endocardial end of the strand to initiate a conditioning excitation wave, and S2 was then applied to a local segment of 0.45 mm on the strand. The exact time window when the unidirectional conduction block occurred was recorded as the VW. Above process was repeated for all locations on the strand to obtain the overall distribution of VWs.

### Measurement of the critical length to the initiation of reentrant spiral waves

The critical length (CL) is the minimal length of the stimulating area for accommodating the evoked spiral wave in a limited tissue size. The CL was measured using the S1–S2 protocol on an idealized 2D sheet model. Similar to the S1–S2 used in 1D simulations, the S1 stimulus was applied to the endocardial side of the tissue sheet to initiate a conditioning wave, and a followed S2 was applied to a local epicardial region during the measured VW. The S2 was always able to evoke a unidirectionally propagated excitation wave, but the wave could evolve into a reentrant spiral wave only when the length of the stimulating region was sufficiently long. The minimum length for a successful initiation of reentrant spiral wave was recorded as the CL.

### Initiation of reentry arrhythmias in the anatomical 2D ventricular slice and 3D ventricle

Reentry arrhythmias in anatomical geometries were initiated using the S1–S2 protocol as well. In the ventricular 2D slice and 3D ventricle, a series of S1 stimuli were applied sequentially to multiple sites on the endocardium as in^67^ to reproduce the activation timing sequence of the Purkinje system^68^. The reentry arrhythmia that took the form of spiral waves in 2D tissue was initiated by a premature stimulus applied to a local slender region on the epicardium during the refractory tail of the previous conditioning wave (i.e., the vulnerable window). Similarly, the reentry arrhythmia (took the form of scroll waves) in the 3D ventricle was also initiated by a premature stimulus, which was applied to the upper part of the left ventricle during the VW.

### Numerical details

The differential equations for the membrane potential and the gating variables were solved by the forward Euler method with a time step of 0.02 ms. For computing state probabilities in the Markov model, the backward Euler method was implemented to avoid using small time step. The spatial resolution was 0.15 mm in 1D strands and idealized 2D tissue sheets. In the realistic 2D ventricular slice and 3D ventricle models, the spatial resolution was set to 0.2 mm to be in line with the anatomical geometry reconstructed from DT-MRI^64,65^. Neumann (non-flux) boundary conditions were adopted to deal with the geometry boundaries^69^.

For solving the differential equations in 1D strand and idealized 2D sheet models, OpenMP parallelization scheme was utilized^70^. For the realistic 2D and 3D models, the optimized GPU algorithm in^71^ was implemented on a Quadro P4000 “Pascal” GPU with 1792 CUDA cores. The host system for the Quadro GPU was a Lenovo ThinkStation P330 with 8 Intel Core i7-9700K CPU cores at 3.6 GHz.

## Supplementary information

Supplementary material

Supplementary video 1

Supplementary video 2

Supplementary video 3

Supplementary video 4

Supplementary video 5

Supplementary video 6

Supplementary information

## Data Availability

All the experimental data used in this study have been referred to in the relevant texts and figure captions. The generated data supporting the conclusion of this article are available within the article and its additional files.

## Code availability

The computer simulation codes used to generate the results in this study can be found at https://github.com/ShugangZhang/T618I.
