# Supplementary material for: Computational analysis of arrhythmogenesis in KCNH2 T618I mutation-associated short QT syndrome and the pharmacological effects of quinidine and sotalol
Source: NPJ Syst Biol Appl. 2022 Nov 4;8:43. doi: 10.1038/s41540-022-00254-5 (PMC9636227; doi:10.1038/s41540-022-00254-5)
Supplement: Supplementary file 1 — Supplementary material [file 41540_2022_254_MOESM1_ESM.pdf]

Supplementary Materials for Computational Analysis of Arrhythmogenesis in *KCNH2* T618I Mutation-Associated Short QT Syndrome and the Pharmacological Effects of Quinidine and Sotalol

Shugang Zhang[1,3], Weigang Lu[2,3,*], Fei Yang[4], Zhen Li[5], Shuang Wang[6], Mingjian Jiang[7], Xiaofeng Wang[8], Zhiqiang Wei[1]

npj Systems Biology and Applications

[1] College of Computer Science and Technology, Ocean University of China, Qingdao 266100, China

[2] Department of Educational Technology, Ocean University of China, Qingdao 266100, China

[3] Biological Physics Group, School of Physics and Astronomy, The University of Manchester, Manchester M13 9PL, U.K.

[4] School of Mechanical, Electrical, and Information Engineering, Shandong University, Weihai 264200, China

[5] College of Computer Science and Technology, Qingdao University, Qingdao 266071, China

[6] College of Computer Science and Technology, China University of Petroleum (East China), Qingdao 266580, China

[7] School of Information and Control Engineering, Qingdao University of Technology, Qingdao 266033, China

[8] MindRank AI ltd., Hangzhou, Zhejiang 311113, China

*: corresponding author, luweigang@ouc.edu.cn

This PDF file includes:

Supplementary Notes 1 - 4

Supplementary Tables 1 - 3

Supplementary Figures 1 - 19

Supplementary References

1 Supplementary Notes

Supplementary Note 1. Simulation of the ECG in wild-type and T618I mutation conditions

The changes of ECG by the T618I mutation were simulated using a transmural ventricular 1D strand. Supplementary Figure 2 plotted the comparison of wild-type and SQT1 pseudo-ECGs, and panel A and B were separately obtained from the homogeneous (EPI:MID:ENDO=1:1:1) and heterogeneous I_{Kr} (EPI:MID:ENDO=1.6:1:1) settings. Our simulation suggested that the incorporation of I_{Kr} heterogeneity was critical in generating the characteristic ECG morphology observed in SQT1 patients, including the abbreviated QT interval and the increased T-wave amplitude. The critical role of I_{Kr} heterogeneity in shaping the T-wave morphology was due to that a larger I_{Kr} conductance in EPI cells led to a greater reduction of epicardial APD comparing to the homogeneous condition, which resulted in a higher membrane potential gradient.

Supplementary Note 2. Actions of quinidine on the ECG of *KCNH2* T618I mutation

Actions of quinidine on the ECG were simulated using the transmural strand with the setting of heterogeneous I_{Kr}. The results are shown in Supplementary Figure 3, where it can be observed that the QT interval was significantly increased, and the peak amplitude of T-wave was reduced. In terms of the QTc, the abbreviated QTc of 315 ms was prolonged to 382 ms, which is close to that in the wild-type condition (400 ms).

Supplementary Note 3. Simulation results based on the ORd model

The ORd model [1] is a widely used human ventricular cell model that has been validated on extensive experimental data from undiseased human ventricular myocytes. Some critical physiological parameters like intracellular ion

concentrations and rate dependence of APD and Ca^{2+} were quantitatively reproduced. It was also designated as the cell model in the Comprehensive in Vitro Proarrhythmia Assay (CiPA) initiative [2]. Therefore, we adopted this model for the model-dependency assessment. Noted that the original I_{Na} in the ORd model was replaced by that in TNNP06 to overcome the unphysiologically slow conduction velocity [3].

- *Simulation results of action potentials*

The developed Markov model was incorporated into the ORD cell model. Simulated action potentials, I_{Kr}, and the statistical data regarding transmural properties under different conditions are plotted in Supplementary Figure 6.

According to the simulation results demonstrated in Supplementary Figure 6, the ORd model successfully reproduced the enhanced I_{Kr} and the abbreviated APD under the mutation condition, and quinidine at 5 μmol was able to restore the APD. Most results are consistent with that using the TNNP06 model (see Figure 7 in the main text for comparison), except that the transmural difference of APD and ERP between ENDO and MID cells were slightly increased in the case of ORd. The detailed action potential parameters under different conditions are listed in Supplementary Table 2.

- *Simulation results of restitution properties and the rate adaption of QT interval*

Simulation results of restitution properties and the rate adaption of the QT interval are shown in Supplementary Figure 7. The main findings based on the ORd model were consistent with that based on the TNNP06 model, such as the flattened/re-steepened APD restitution curves under the mutation/quinidine condition, the decreased CV after the application of quinidine, and the flattened/restored QT rate adaption under the mutation/quinidine condition (see Figure 9 in the main text for comparison).

- *Simulation results of pseudo-ECGs*

Simulation results of pseudo-ECGs using 1D strand of ORd cells are shown in Supplementary Figure 8. As observed in the figure, the model recapitulated the abbreviated QT interval under the mutation condition, and quinidine successfully restored the QT interval to its WT level. However, the model did not reproduce the increased amplitude of T-wave under the mutation condition (see Supplementary Figure 3 in this document for comparison).

- *Simulation results of the temporal vulnerability to reentry arrhythmias*

Simulation results of the temporal vulnerable window based on the ORd model was demonstrated in Supplementary Figure 9. The average width of VWs was decreased from 1.48 ms under the control condition to 0.48 ms under T618I. The conclusion that the overall temporal vulnerability is decreased in the mutation therefore remain unchanged.

- *Simulation results of the spatial vulnerability to reentry arrhythmias*

Simulation of the spatial critical length (CL) to reentry arrhythmias based on the ORd model was shown in Supplementary Figure 10. The measured CLs were 52.7 and 44.4 mm in the wild-type and T618I tissue, separately, suggesting an increased spatial vulnerability. Quinidine significantly restored the critical length (64.7 mm). Above observations are consistent with that on the TNNP06 model.

Supplementary Note 4. Simulation results based on the ToR-ORd model

ToR-ORd [4] is another well-calibrated human ventricular cell model. It was derived from the original ORd model and was further validated under various conditions including health, disease, and drug blockade. According to the original report of ToR-ORd, the model behaviour was consistent with a wide range of established biophysical knowledge and experimental data. Therefore, the ToR-ORd is also a suitable model for the model-dependency assessment of our findings

in this study.

- *Simulation results of action potentials*

We first conducted cellular level simulations using the ToR-ORd model. Similar to the previous two models, the developed Markov model was incorporated into the cell model. Simulated action potentials, I_{Kr}, and the statistical data regarding transmural properties under different conditions are plotted in Supplementary Figure 11.

According to the simulation results demonstrated in Supplementary Figure 11, the main findings at the cellular level remain unchanged (see Figure 7 in the main text for comparison). Detailed action potential parameters under different conditions are listed in Supplementary Table 3.

- *Simulation results of restitution properties and the rate adaption of QT interval*

Simulation results of restitution properties and the rate adaption of the QT interval are shown in Supplementary Figure 12. The main findings based on the TNNP06 model were consistent with that based on the ToR-ORd model, such as the flattened/re-steepened APD restitution curves under the mutation/quinidine condition, the decreased CV after the application of quinidine, and the flattened/restored QT rate adaption under the mutation/quinidine condition (see Figure 9 in the main text for comparison).

- *Simulation results of pseudo-ECGs*

Simulation results of pseudo-ECGs using 1D strand of the ToR-ORd cells are shown in Supplementary Figure 13. As observed in the figure, the model successfully recapitulated the abbreviated/restored QT interval under the mutation/quinidine condition. However, similar to the ORd model, ToR-ORd also failed to reproduce the increased amplitude of T-wave under the mutation condition.

- *Simulation results of the temporal vulnerability to reentry arrhythmias*

Simulation results of the temporal vulnerable window based on the ORd model was demonstrated in Supplementary Figure 14. The average width of VWs was decreased from 4.62 ms under the control condition to 4.06 ms under T618I. The overall temporal vulnerability is decreased in the mutation.

- *Simulation results of the spatial vulnerability to reentry arrhythmias*

Simulation of the spatial critical length (CL) to reentry arrhythmias based on the ToR-ORd model was shown in Supplementary Figure 15. The measured CLs were 57.0 and 37.1 mm in the wild-type and T618I tissue, separately, suggesting an increased spatial vulnerability. Quinidine significantly restored the critical length (66.0 mm). Above observations are consistent with that on the TNNP06 model.

Above all, the simulation results are highly consistent among various models except that the increased T-wave amplitude was only reproduced in TNNP06. Therefore, cautions should be taken when interpreting the results of increased T-wave amplitude generated by the TNNP06 model. Despite the above inconsistency, most simulation results reported in this paper are robust and are not model dependent.

2 Supplementary Tables

Supplementary Table 1: Action potential parameters for wild-type and T618I conditions.

	Wild-type			*KCNH2* T618I			Quinidine		
	EPI	ENDO	MID	EPI	ENDO	MID	EPI	ENDO	MID
APD_{25}	210.46	210.34	286.06	149.18	158.22	190.68	169.16	180.02	228.60
APD_{50}	276.16	283.84	374.12	203.12	220.84	257.96	235.36	252.22	326.30
APD_{90}	308.94	319.98	411.66	235.16	255.76	295.46	284.74	304.22	388.22
ERP	356.94	354.98	457.66	280.16	287.76	340.46	318.74	335.22	424.22
V_{rest}	-85.47	-85.46	-85.30	-85.62	-85.60	-85.50	-85.23	-85.19	-84.95
$\mathrm{d}V/\mathrm{dt}_{\max}$	387.23	387.86	386.77	390.81	390.62	387.35	332.32	332.06	328.60
OS	41.54	42.69	41.44	41.53	42.71	41.39	36.50	37.78	36.21

Supplementary Table 2: Action potential parameters using the ORd model.

	Wild-type			*KCNH2* T618I			Quinidine		
	EPI	ENDO	MID	EPI	ENDO	MID	EPI	ENDO	MID
APD_{25}	166.44	170.36	191.58	95.56	92.58	116.04	112.74	99.50	148.08
APD_{50}	226.76	241.36	256.40	127.94	130.24	147.54	166.94	190.62	218.62
APD_{90}	270.74	296.94	304.70	177.74	189.14	199.50	269.74	328.22	347.88
ERP	284.74	310.94	316.70	191.74	204.14	211.50	283.74	342.22	347.88
V_{rest}	-87.86	-87.97	-87.58	-87.93	-88.02	-87.71	-88.01	-88.07	-87.80
$\mathrm{d}V/\mathrm{dt}_{\text{max}}$	448.02	456.07	429.78	447.99	456.61	429.24	386.49	392.89	370.63
OS	43.65	44.90	42.19	43.43	44.79	41.67	38.58	39.67	37.26

Supplementary Table 3: Action potential parameters using the ToR-ORd model.

	Wild-type			*KCNH2* T618I			Quinidine		
	EPI	ENDO	MID	EPI	ENDO	MID	EPI	ENDO	MID
APD_{25}	136.26	138.66	141.06	81.48	86.18	95.02	87.74	90.04	97.04
APD_{50}	202.98	229.02	242.86	110.98	129.38	136.36	140.58	168.92	205.96
APD_{90}	244.52	287.54	302.22	155.68	189.58	207.60	234.18	283.78	323.00
ERP	259.52	302.54	314.22	170.68	205.58	220.60	250.18	300.78	337.00
V_{rest}	-89.38	-89.03	-89.39	-89.21	-88.87	-89.54	-89.31	-89.06	-90.03
$\mathrm{d}V/\mathrm{dt}_{\mathrm{max}}$	337.30	345.86	316.76	343.72	350.28	317.40	269.41	279.93	241.95
OS	35.17	35.36	33.24	35.61	35.78	34.19	30.40	30.35	30.19

3 Supplementary Figures

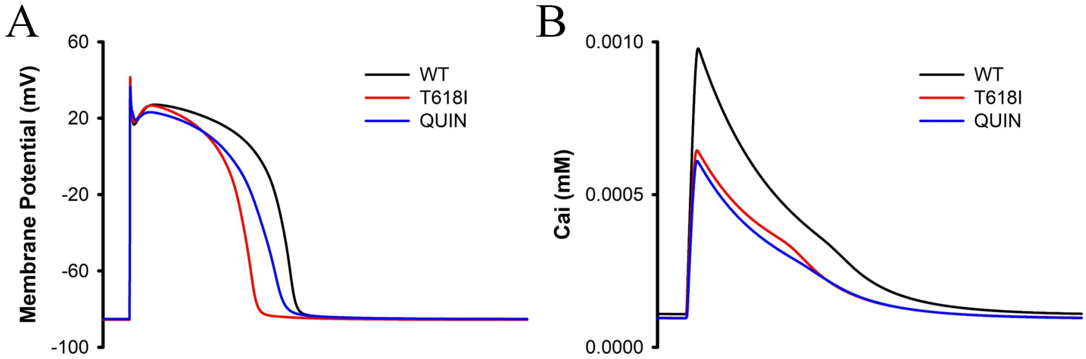

Supplementary Figure 1: Simulation results of calcium transients under different conditions. (A) Action potentials. (B) Calcium transients. Simulations demonstrated that the intracellular Ca^{2+} concentration was decreased under the T618I condition, and quinidine did not cause obvious effects.

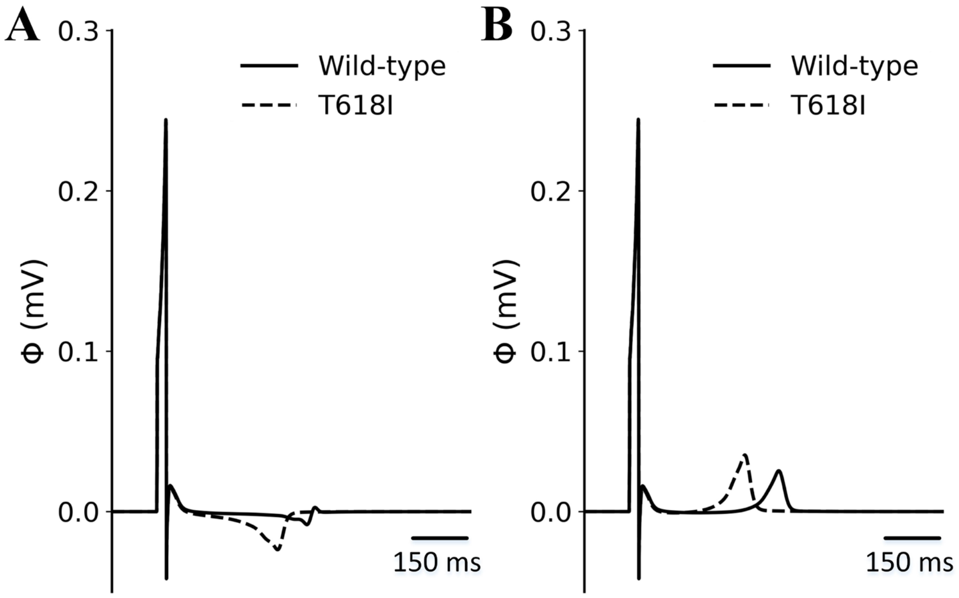

Supplementary Figure 2: Simulation results of pseudo-ECGs using the TNNP06 model. (A) Homogeneous I_{Kr} setting, i.e., EPI:MID:ENDO=1:1:1. (B) Heterogeneous I_{Kr} setting, i.e., EPI:MID:ENDO=1.6:1:1. Solid and dashed lines represent the wild-type and T618I conditions, respectively.

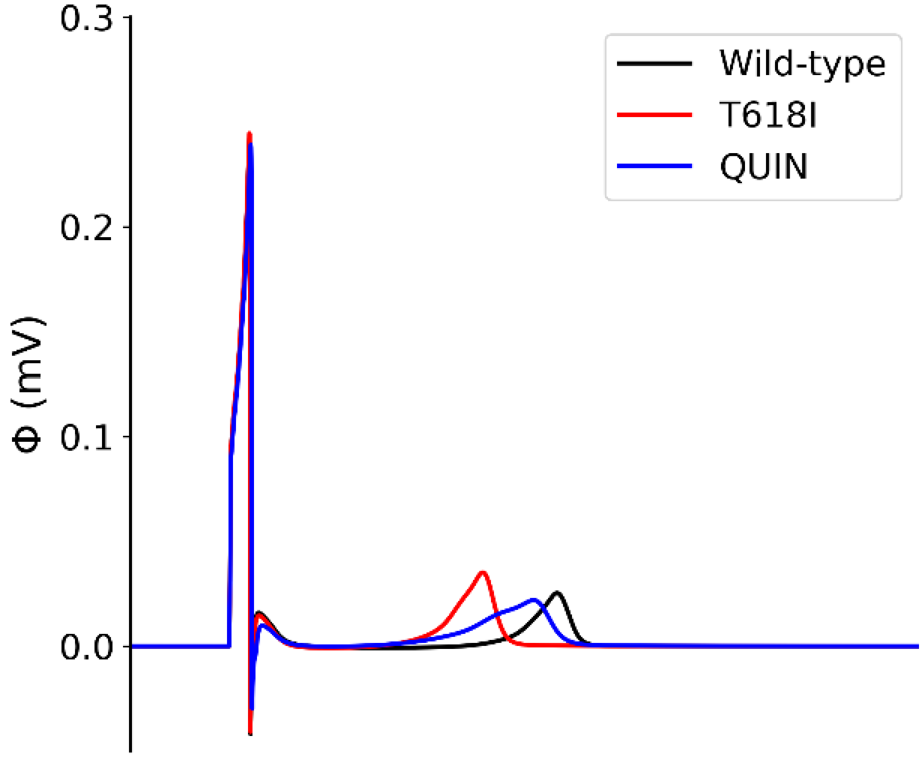

Supplementary Figure 3: Simulation results of pseudo-ECGs in wild-type, T618I, and quinidine conditions. Quinidine successfully restored the abbreviated QT interval.

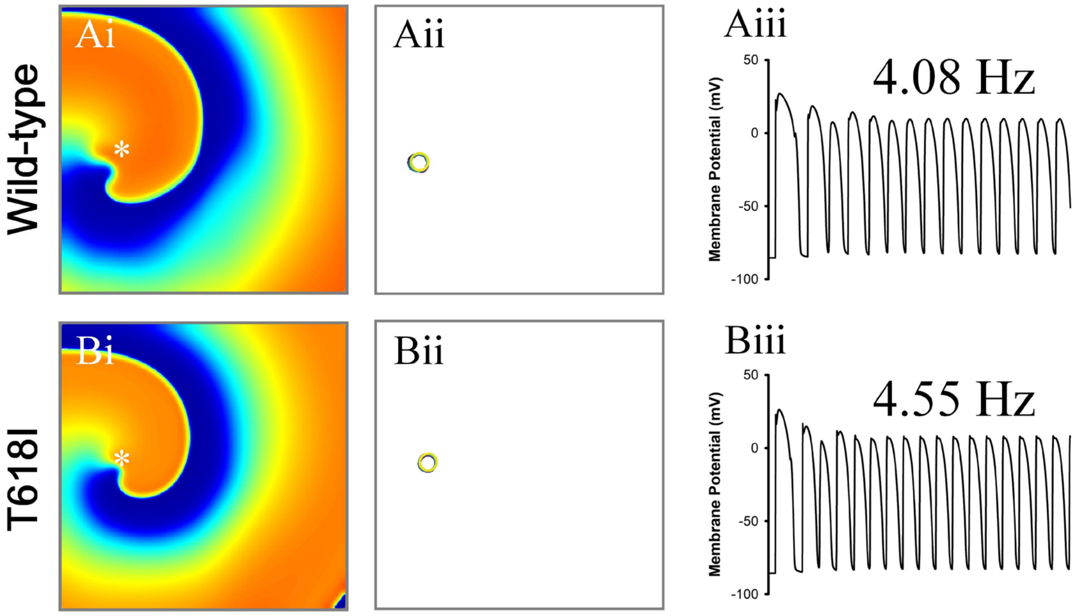

Supplementary Figure 4: Effects of the KCNH2 T618I mutation on the dynamic behaviour of reentrant spiral waves. (A) Wild-type, (B) mutation conditions. Panels from left to right show the spiral wave form, the tip trajectory, and the action potential trace of the representative cell marked '*', respectively.

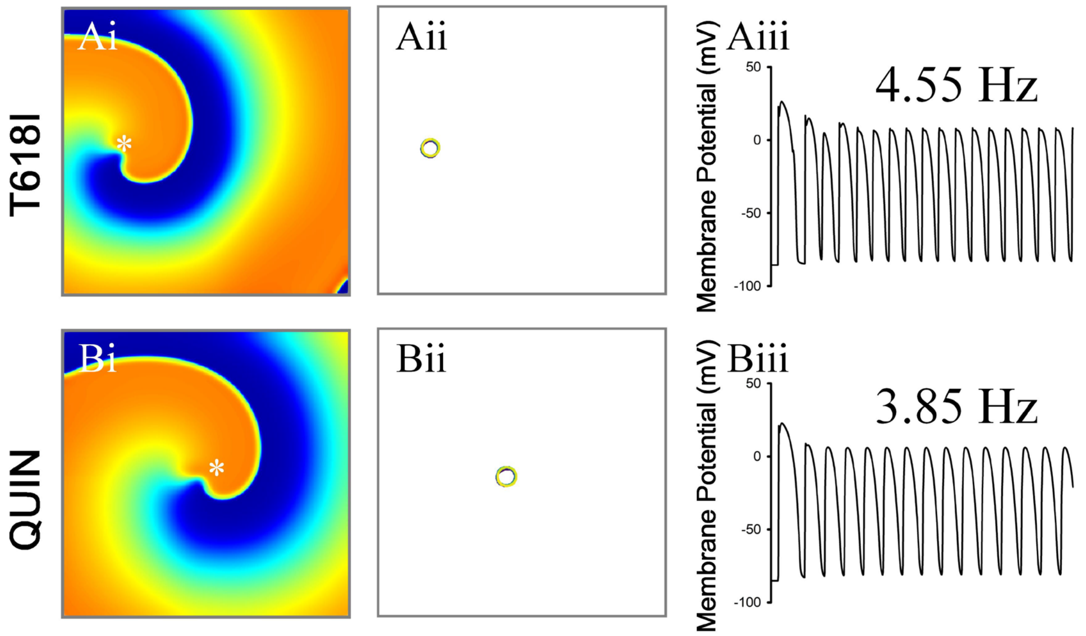

Supplementary Figure 5: Actions of quinidine on the dynamic behaviour of reentrant spiral waves. (A) The mutation condition, (B) with the administration of quinidine. Panels from left to right show the spiral wave form, the tip trajectory, and the action potential trace of the representative cell marked '*', respectively.

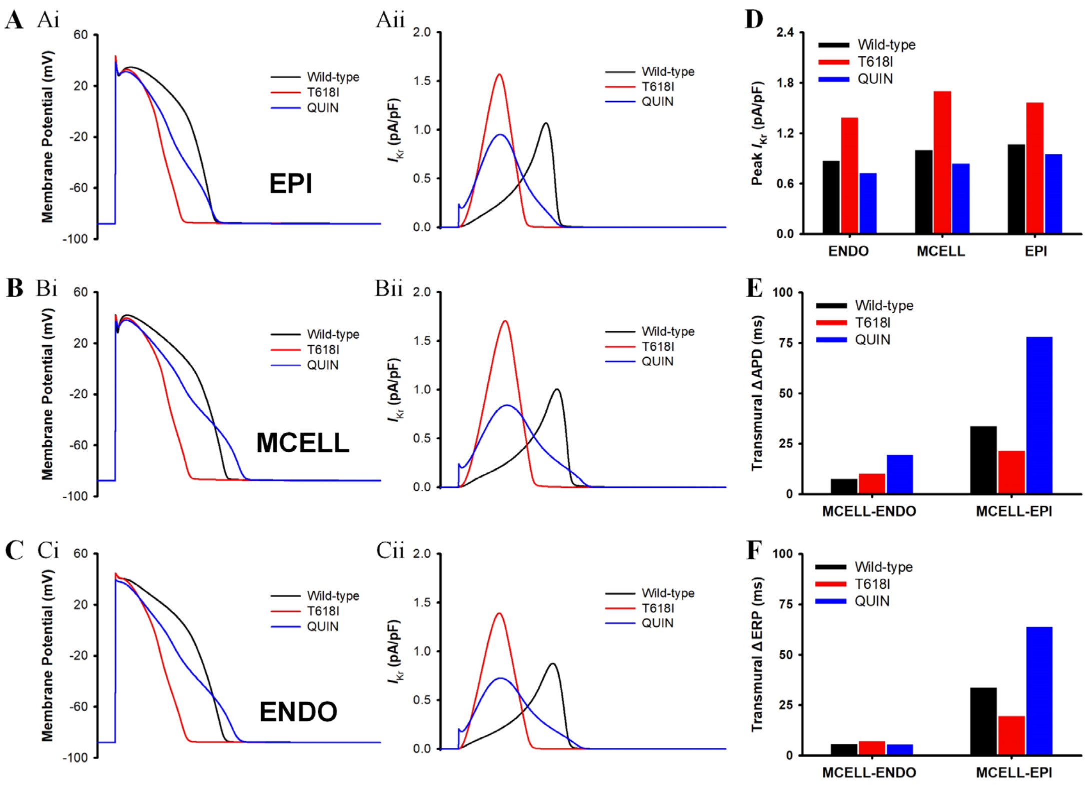

Supplementary Figure 6: Simulation results using the ORd model. (A-C) Steady-state (1.25 Hz) action potentials and the corresponding I_{Kr} for EPI, MID, and ENDO cells. (D) The comparison of the peak I_{Kr}. (E) The comparison of transmural APD differences. (F) The comparison of transmural ERP differences.

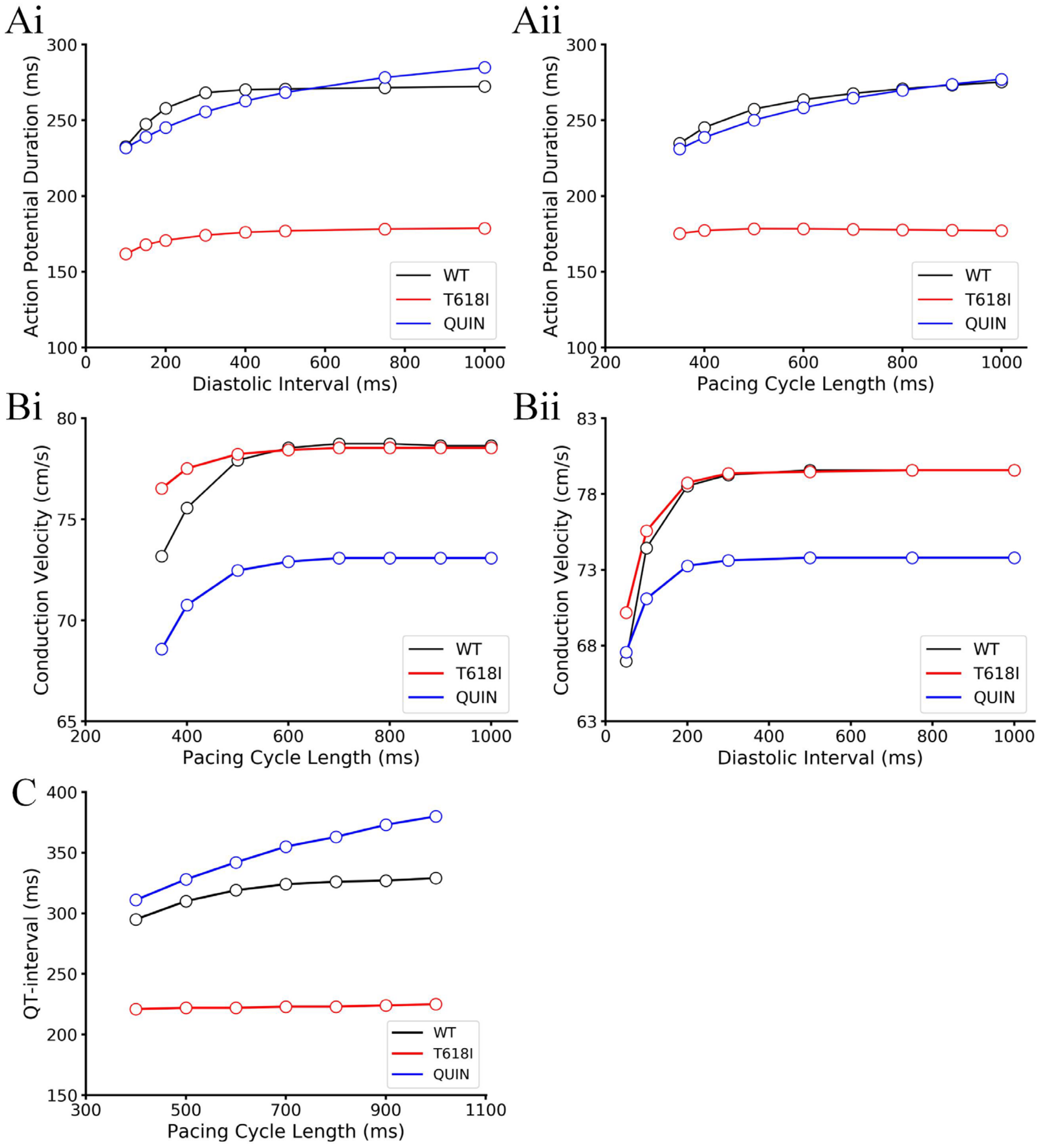

Supplementary Figure 7: Simulation results of APD and CV restitution curves and the rate adaption curves of QT interval using the ORd model. (A) APD restitution curves obtained from the S1-S2 protocol (Ai) and the dynamic protocol (Aii), where APDs were plotted against PCL and DI, respectively. (B) CV restitution curves obtained from the S1-S2 protocol (Bi) and the dynamic protocol (Bii). (C) The rate adaption curves of QT interval.

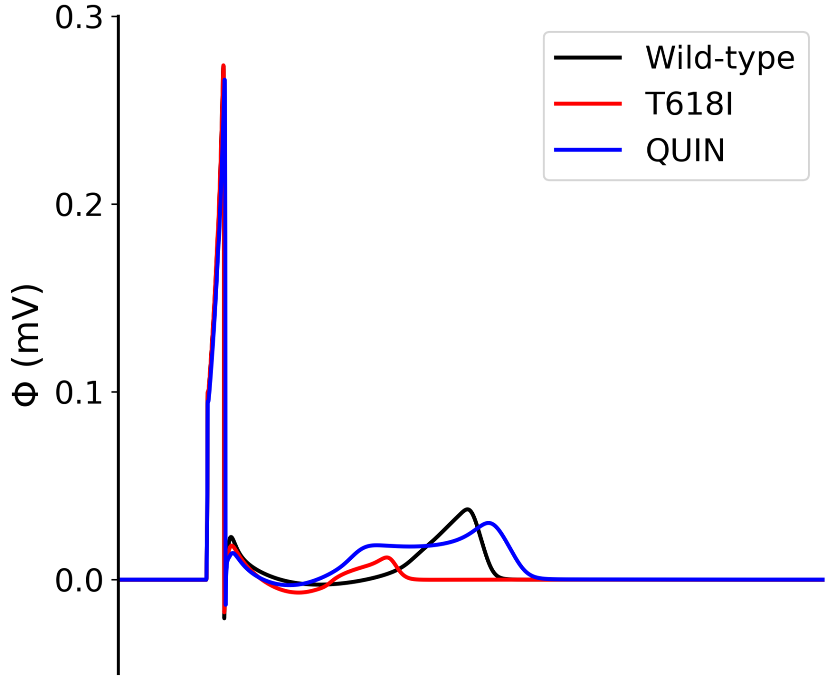

Supplementary Figure 8: Simulation results of pseudo-ECGs using the ORd model.

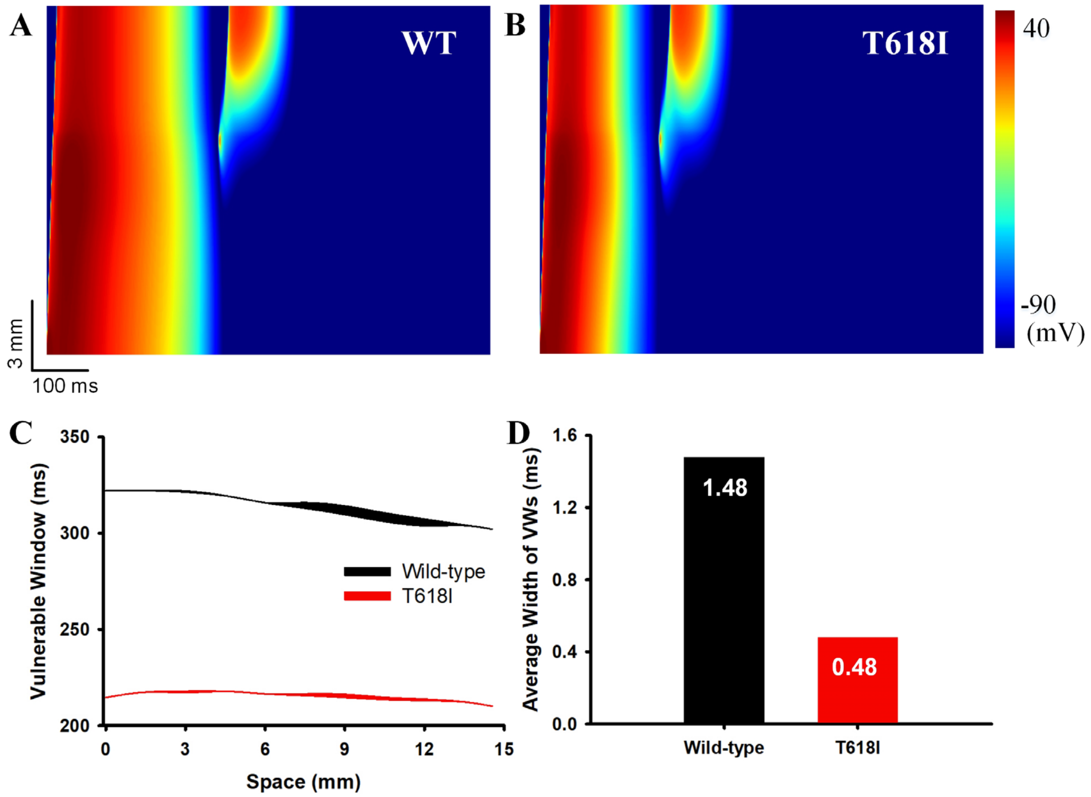

Supplementary Figure 9: Simulation results of the unidirectional conduction block based on the ORd model. (A) The evoked unidirectional conduction block in the wild-type condition. (B) The unidirectional conduction block in the T618I mutation condition. (C) Distributions of VWs in the two conditions. (D) The comparison of the average VW width in the two conditions.

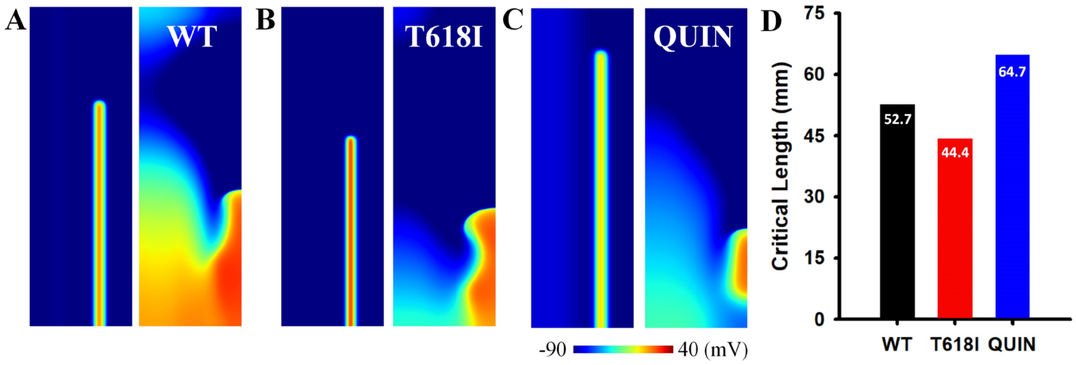

Supplementary Figure 10: Simulation results of the critical length based on the ORd model. (A) Wild-type, (B) T618I mutation, and (C) quinidine conditions. (D) The comparison of critical length in the above situations.

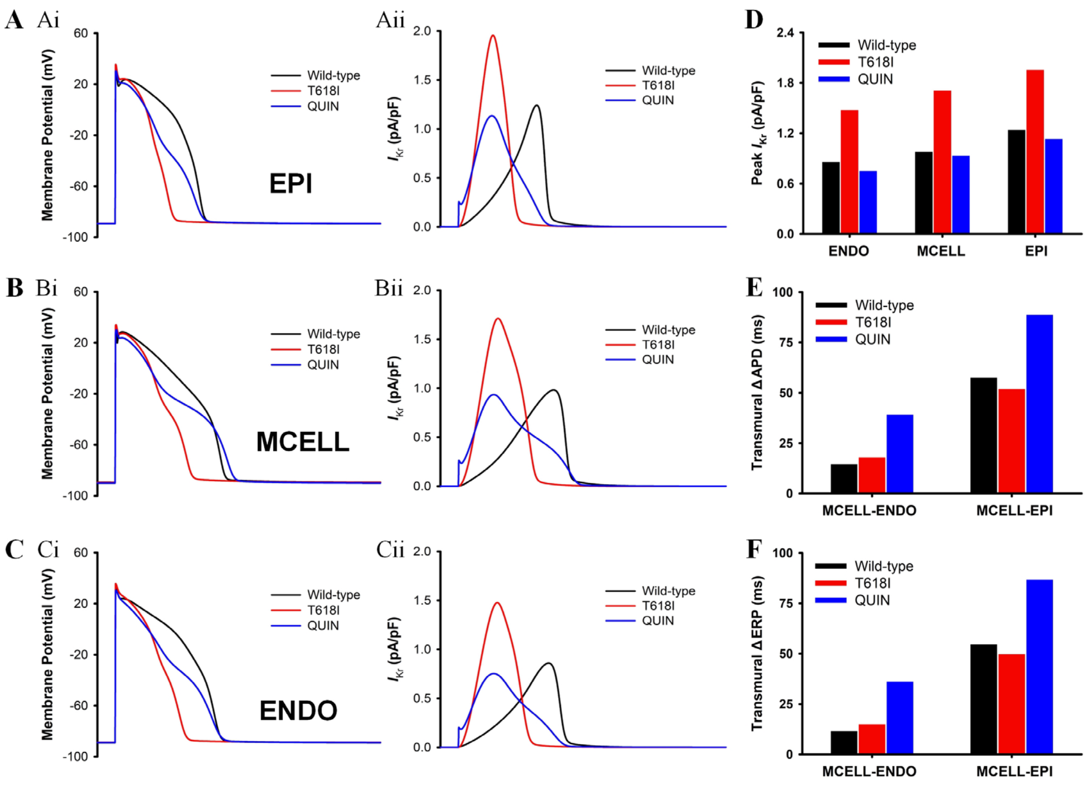

Supplementary Figure 11: Simulation results using the ToR-ORd model. (A-C) Steady-state (1.25 Hz) action potentials and the corresponding I_{Kr} for EPI, MID, and ENDO cells. (D) The comparison of the peak I_{Kr}. (E) The comparison of transmural APD differences. (F) The comparison of transmural ERP differences.

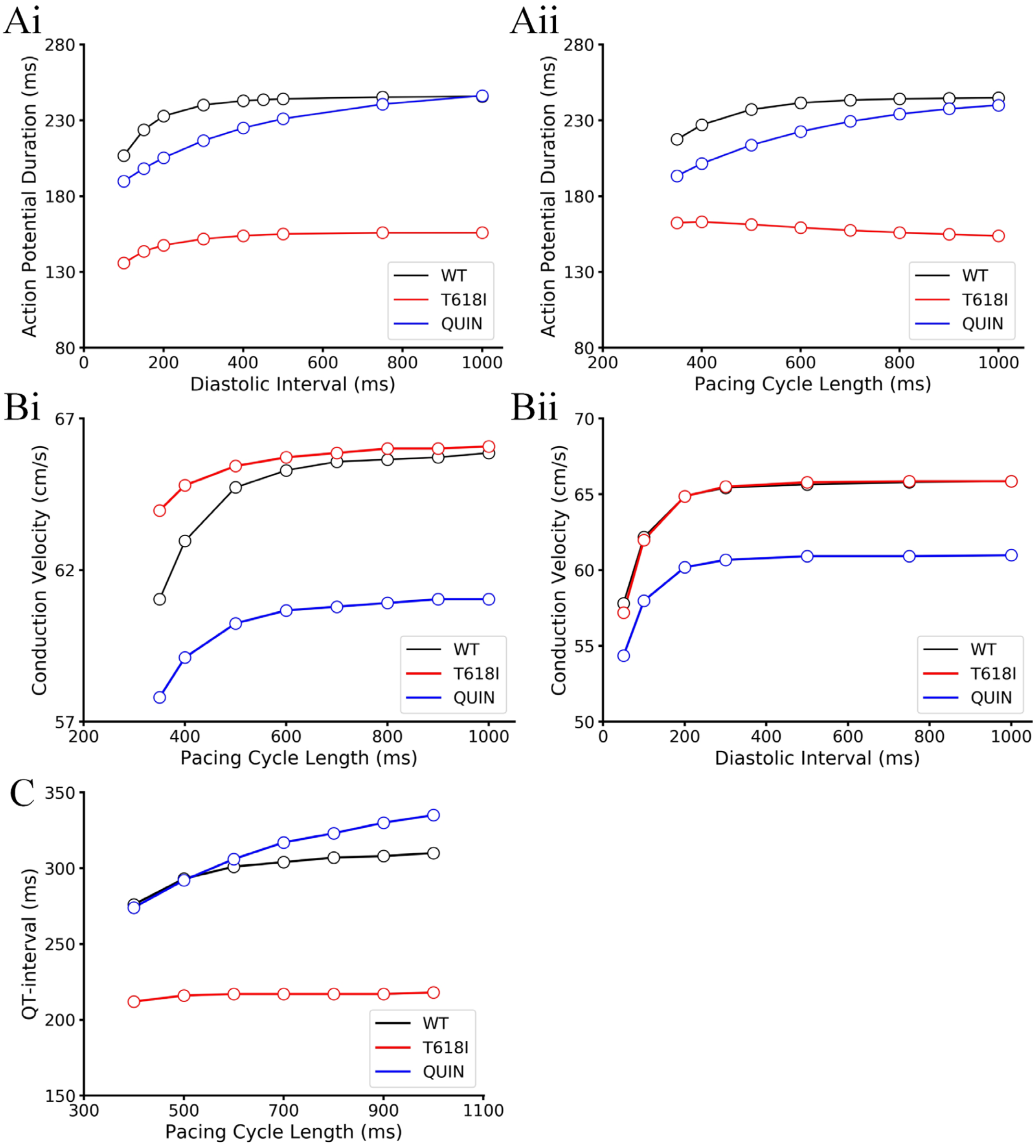

Supplementary Figure 12: Simulation results of APD and CV restitution curves and the rate adaption curves of QT interval using the ToR-ORd model. (A) APD restitution curves obtained from the S1-S2 protocol (Ai) and the dynamic protocol (Aii), where APDs were plotted against PCL and DI, respectively. (B) CV restitution curves obtained from the S1-S2 protocol (Bi) and the dynamic protocol (Bii). (C) The rate adaption curves of QT interval.

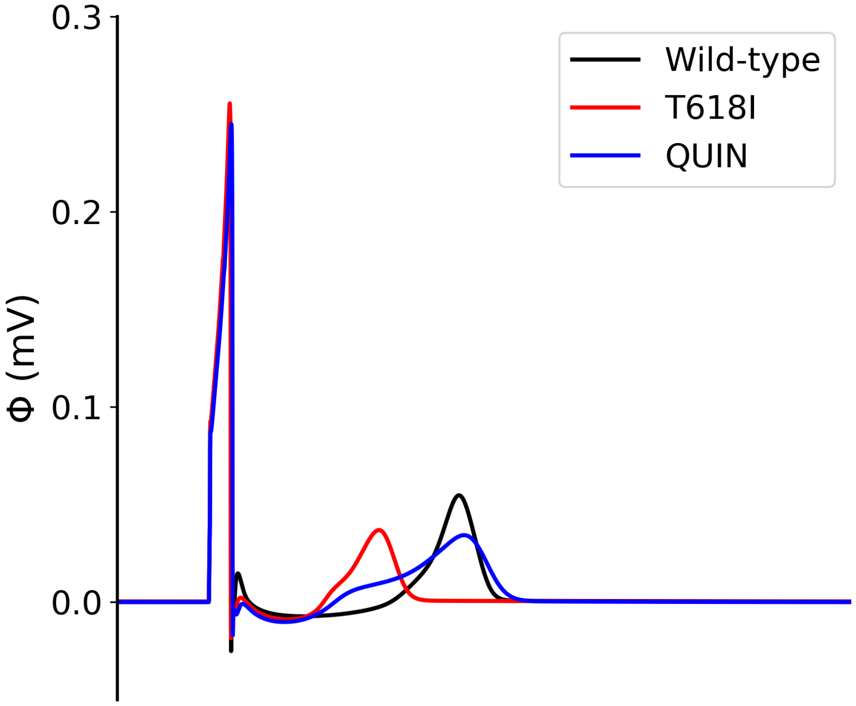

Supplementary Figure 13: Simulation results of pseudo-ECGs using the ToR-ORd model.

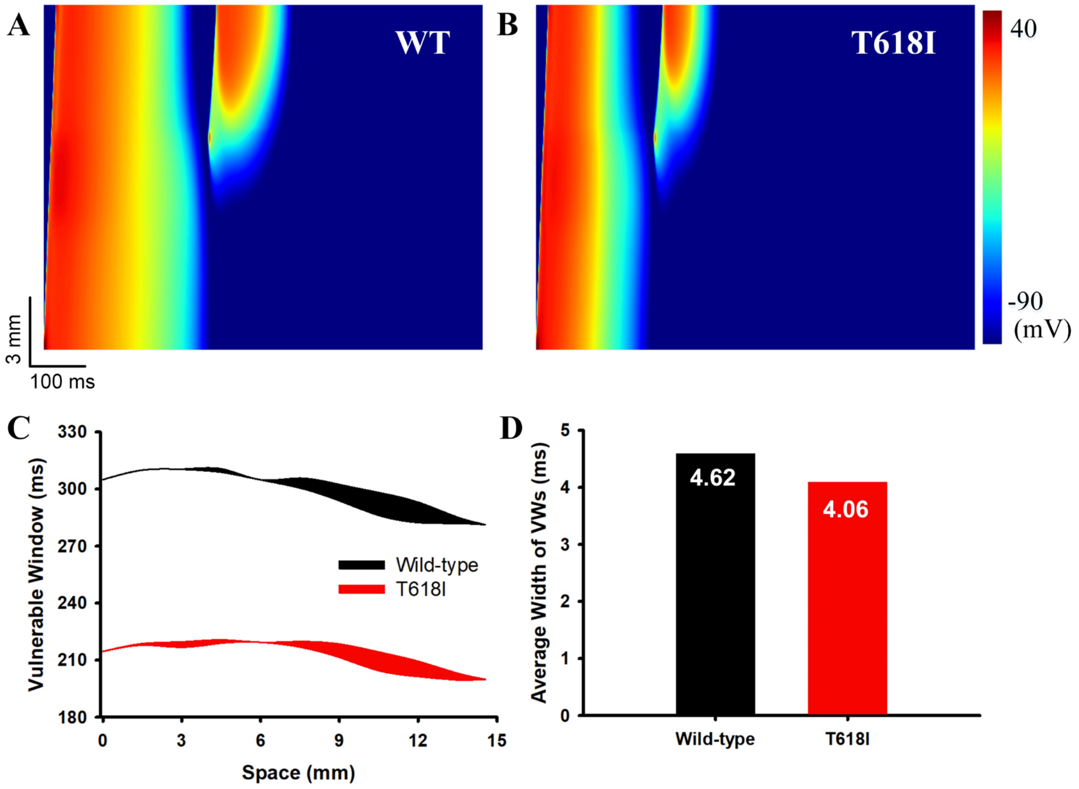

Supplementary Figure 14: Simulation results of the unidirectional conduction block based on the ToR-ORd model. (A) The evoked unidirectional conduction block in the wild-type condition. (B) The unidirectional conduction block in the T618I mutation condition. (C) Distributions of VWs in the two conditions. (D) The comparison of the average VW width in the two conditions.

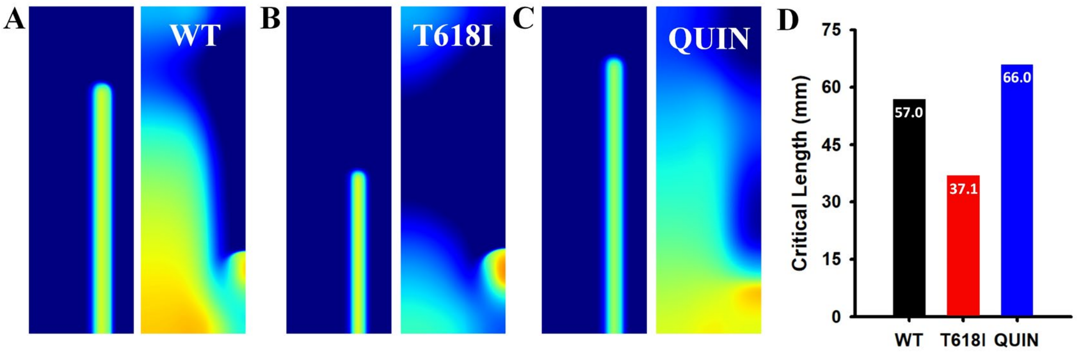

Supplementary Figure 15: Simulation results of the critical length based on the ToR-ORd model. (A) Wild-type, (B) T618I mutation, and (C) quinidine conditions. (D) The comparison of critical length in the above situations.

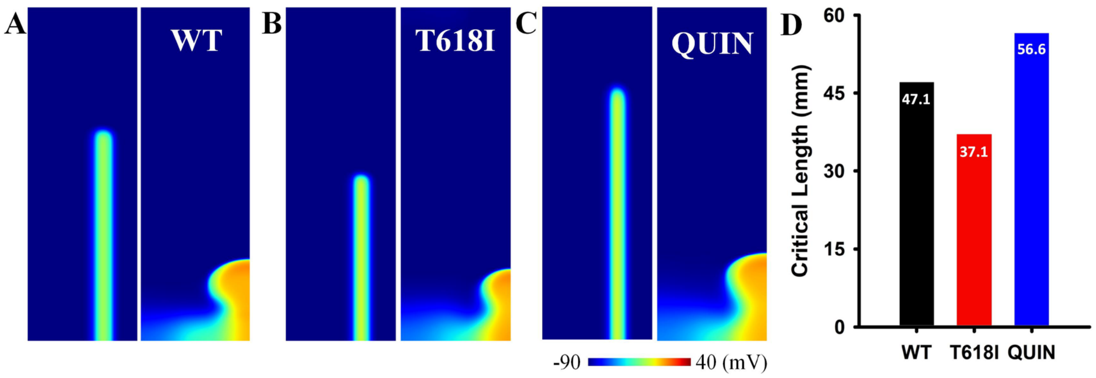

Supplementary Figure 16: Simulation results of the critical length based on the TNNP06 model. (A) Wild-type, (B) T618I mutation, and (C) quinidine conditions. (D) The comparison of critical length in the above situations.

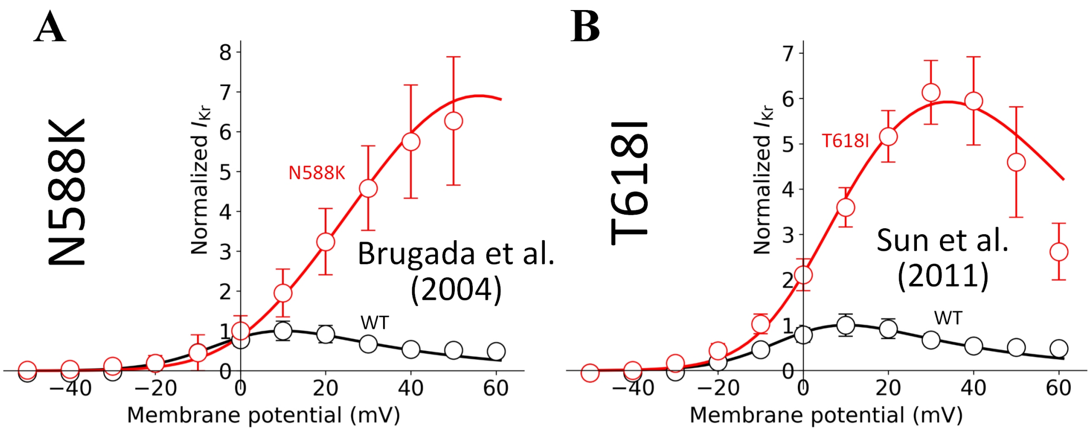

Supplementary Figure 17: Simulated I-V curves along with the experimental data. (A) N588K. Experimental data from [5]; (B) T618I. Experimental data from [6]. Error bars in this figure represent standard error of measurement (SEM).

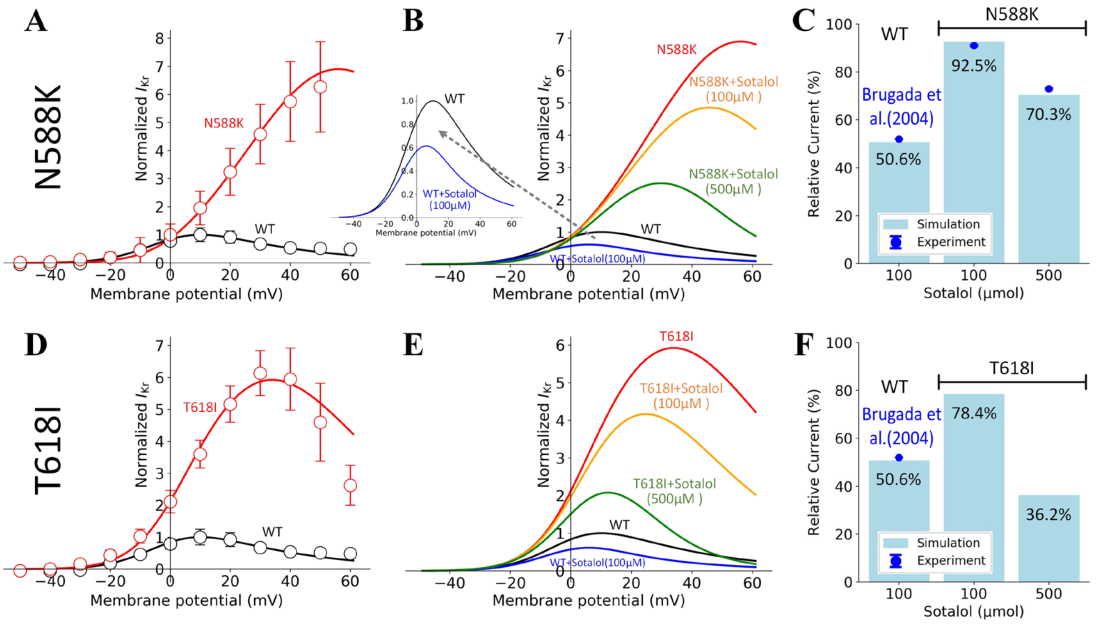

Supplementary Figure 18: Simulation results of the I-V curves under different conditions. (A) The fitted current-voltage (I-V) curves in wild-type (black) and N588K (red) conditions. Experimental data of N588K is obtained from [6]. (B) The simulated I-V curves after the application of 100 or 500 µmol sotalol under wild-type and N588K conditions. The inset shows the comparison of I-V curves under the control condition. (C) The relative N588K I_{Kr} current at 20 mV after the application of sotalol. Experimental data from [5]. (D) The fitted current-voltage (I-V) curves in wild-type (black) and T618I (red) conditions. Experimental data from [5]. (E) The simulated I-V curves after the application of 100 or 500 µmol sotalol under wild-type and N588K conditions. (F) The relative T618I I_{Kr} current at 20 mV after the application of sotalol. Error bars in this figure represent standard error of measurement (SEM).

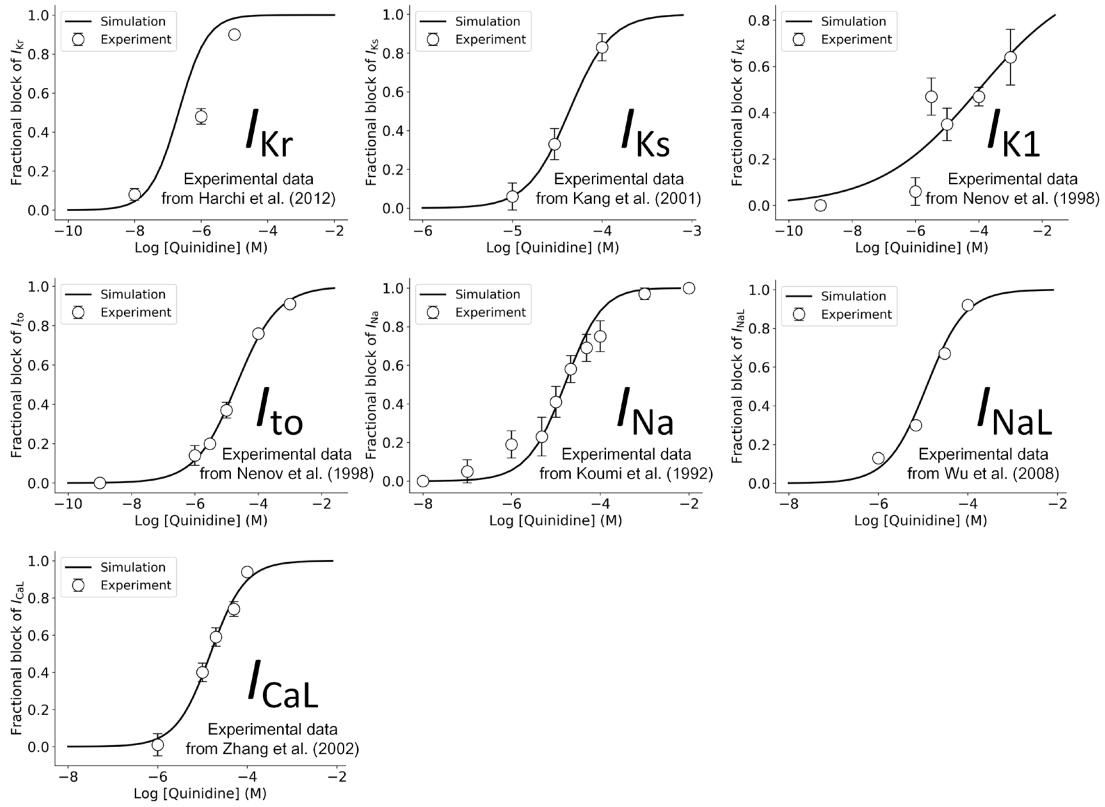

Supplementary Figure 19: Dose-dependent effects of quinidine on multiple ion channels. Experimental data sources: I_{Kr} from Harchi et al. (Fig. 7B in [7]); I_{Ks} from Kang et al. (Fig. 2B in [8]); I_{K1} from Nenov et al. (Fig. 4C in [9]); I_{to} from Nenov et al. (Fig. 1B in [9]); I_{Na} from Koumi et al. (Fig. 2 in [10]); I_{NaL} from Wu et al. (Fig. 5D in [11]); I_{CaL} from Zhang et al. (Fig. 1f in [12]). Error bars in this figure represent the standard error of measurement (SEM).

Supplementary References

1. O'Hara T, Virág L, Varró A, Rudy Y. Simulation of the undiseased human cardiac ventricular action potential: model formulation and experimental validation. PLoS Comput Biol. 2011;7:e1002061.
2. Colatsky T, Fermini B, Gintant G, Pierson JB, Sager P, Sekino Y, et al. The Comprehensive in Vitro Proarrhythmia Assay (CiPA) initiative — Update on progress. J Pharmacol Toxicol Methods. 2016;81:15–20.
3. Elshrif MM, Cherry EM. A quantitative comparison of the behavior of human ventricular cardiac electrophysiology models in tissue. PLoS One. 2014;9:e84401.
4. Tomek J, Bueno-Orovio A, Passini E, Zhou X, Minchole A, Britton O, et al. Development, calibration, and validation of a novel human ventricular myocyte model in health, disease, and drug block. Elife. 2019;8.
5. Brugada R, Hong K, Dumaine R, Cordeiro J, Gaita F, Borggrefe M, et al. Sudden Death Associated with Short-QT Syndrome Linked to Mutations in HERG. Circulation. 2004;109:30–5.
6. Sun Y, Quan XQ, Fromme S, Cox RH, Zhang P, Zhang L, et al. A novel mutation in the KCNH2 gene associated with short QT syndrome. J Mol Cell Cardiol. 2011;50:433–41.
7. El Harchi A, Melgari D, Zhang YH, Zhang H, Hancox JC. Action Potential Clamp and Pharmacology of the Variant 1 Short QT Syndrome T618I hERG K+ Channel. PLoS One. 2012;7.
8. Kang J, Chen XL, Wang L, Rampe D. Interactions of the antimalarial drug mefloquine with the human cardiac potassium channels KvLQT1/minK and HERG. J Pharmacol Exp Ther. 2001;299:290–6.
9. Nenov NI, Crumb WJ, Pigott JD, Harrison LH, Clarkson CW. Quinidine interactions with human atrial potassium channels developmental aspects. Circ Res. 1998;83:1224–31.
10. Koumi SI, Sato R, Katori R, Hisatome I, Nagasawa K, Hayakawa H. Sodium channel states control binding and unbinding behaviour of antiarrhythmic drugs in cardiac myocytes from the Guinea pig. Cardiovasc Res. 1992;26:1199–205.
11. Wu L, Guo D, Li H, Hackett J, Yan GX, Jiao Z, et al. Role of late sodium current in modulating the proarrhythmic and antiarrhythmic effects of quinidine. Hear Rhythm. 2008;5:1726–34.
12. Zhang YH, Hancox JC. Mode-dependent inhibition by quinidine of NA+-CA2+ exchanger

current from guinea-pig isolated ventricular myocytes. Clin Exp Pharmacol Physiol. 2002;29:777–81.
