# Supplementary material for: Computational analysis of arrhythmogenesis in KCNH2 T618I mutation-associated short QT syndrome and the pharmacological effects of quinidine and sotalol
Source: NPJ Syst Biol Appl. 2022 Nov 4;8:43. doi: 10.1038/s41540-022-00254-5 (PMC9636227; doi:10.1038/s41540-022-00254-5)
Supplement: Supplementary file 8 — Supplementary information [file 41540_2022_254_MOESM8_ESM.docx]

# Additional Files

**Supplementary_Material.docx** Additional methods and simulation results.

**Supplementary_Video_1.avi** WT reentry in realistic 2D slice.

**Supplementary_Video_2.avi** T618I reentry in realistic 2D slice.

**Supplementary_Video_3.avi** Quinidine reentry in realistic 2D slice.

**Supplementary_Video_4.avi** WT reentry in realistic 3D ventricle.

**Supplementary_Video_5.avi** T618I reentry in realistic 3D ventricle.

**Supplementary_Video_6.avi** Quinidine reentry in realistic 3D ventricle.
